# Supplementary material for: Estimating the resilience of non-stationary systems
Source: Nat Commun. 2026 Nov 6;17:11286. doi: 10.1038/s41467-026-78489-7 (PMC13743781; doi:10.1038/s41467-026-78489-7)
Supplement: Supplementary file 1 [file 41467_2026_78489_MOESM1_ESM.pdf]

Supplement to: Estimating the Resilience of Non-Stationary Systems

Taylor Smith[1,2*], Andreas Morr[3,4], Christof Schötz[2,4], Niklas Boers[2,4]
[1]Institute of Geosciences, Universität Potsdam, Potsdam, Germany
[2]Munich Climate Center and Earth System Modelling Group, Department of Aerospace and Geodesy, TUM School of Engineering and Design, Technical University of Munich, Munich, Germany
[3]Department of Mathematics, School of Computation, Information and Technology, Technical University of Munich, Munich, Germany
[4]Research Domain IV–Complexity Science, Potsdam Institute for Climate Impact Research, Potsdam, Germany

Corresponding author:
Taylor Smith
Email: tasmith@uni-potsdam.de

Synthetic Time Series Models

For the pitchfork-bifurcation time series model, we use the same model setup as was used in Smith et al. (2026). Scripts to reproduce our model code can also be found on the Zenodo archive for this publication (https://doi.org/10.5281/zenodo.19731233).

The parameters used can also be found here:

Parameter	Value / Description
Time step Δt	0.01 days
Simulation duration	1950–2025
Initial condition x_0	0.01
Control parameter range $(p_{\text{start}}, p_{\text{end}})$	$(-0.5, 0.5)$
Noise amplitude σ	0.025
Seasonal amplitude A_{seasonal}	0.05
Seasonal frequency ω	365 day
Integration scheme	Euler–Maruyama
Rescaling range (output)	$[-0.1, 1]$

Supplemental Table S1: Parameters used in the pitchfork bifurcation time series model.

This basic model is used in Figure 1 and Supplemental Figures S1, S8, S9, S12, and S14. We generate additional realizations of this model with different noise forcing for Supplemental Figures S6, S11, and S13, and with additional sub-annual harmonics for Supplemental Figure S7.

For completeness, we generate a second time series describing a saddle-node bifurcation using a cubic fold model with seasonal forcing:

$$\frac{\mathrm{d}x}{\mathrm{d}t} = x - x^3 + p(t) + A\cos(\omega t) + \sigma\,\xi(t) \tag{1}$$

We vary the control parameter p linearly to produce the transition from a high to a low state; we note that our parametric λ estimate remains a concise early warning signal (Supplemental Figure S10). The parameters for the model can be found here:

Parameter	Value / Description
Time step Δt	0.1
Simulation duration	1950–2025
Initial condition x_0	1.0
Control parameter range $(p_{\text{start}}, p_{\text{end}})$	$(0.4, -0.8)$
Noise amplitude σ	0.02
Seasonal amplitude A_{seasonal}	0.05
Seasonal frequency ω	365 day
Integration scheme	Euler–Maruyama

Supplemental Table S2: Parameters used in the cubic fold bifurcation time series model.

Synthetic Vegetation Model

For the reaction-diffusion spatial vegetation model, we use the same model setup as was used in Smith et al. (2026). Scripts to reproduce our model code can be found on Zenodo (https://doi.org/10.5281/zenodo.18031340).

The parameters used can also be found here:

**Parameter**	**Value / Description**
Grid size	50×50
Time step Δt	0.1 days
Simulation duration	20 years
Base growth rate r_v	0.5
Vegetation half-saturation constant h_v	0.2
Environmental half-saturation constant h_E	2.0
Nonlinearity exponent p	4.0
Diffusion coefficient D	0.5
Noise amplitude σ	0.02
Initial environmental forcing $E_{0,\text{start}}$	4.0
Final environmental forcing $E_{0,\text{end}}$	8.0
Seasonal amplitude in E_0 (A_{E_0})	0.15
Mean seasonal amplitude in r_v ($A_{r_v,\text{mean}}$)	0.15
Std. of amplitude heterogeneity ($A_{r_v,\text{std}}$)	0.03
Max spatial phase shift	$\pi/6$ ($\sim$ 30 days)
Seasonal period T_{season}	365 days
Integration scheme	Euler–Maruyama

Supplemental Table S3: Parameters used in the spatial reaction-diffusion vegetation model.

Supplemental Figures

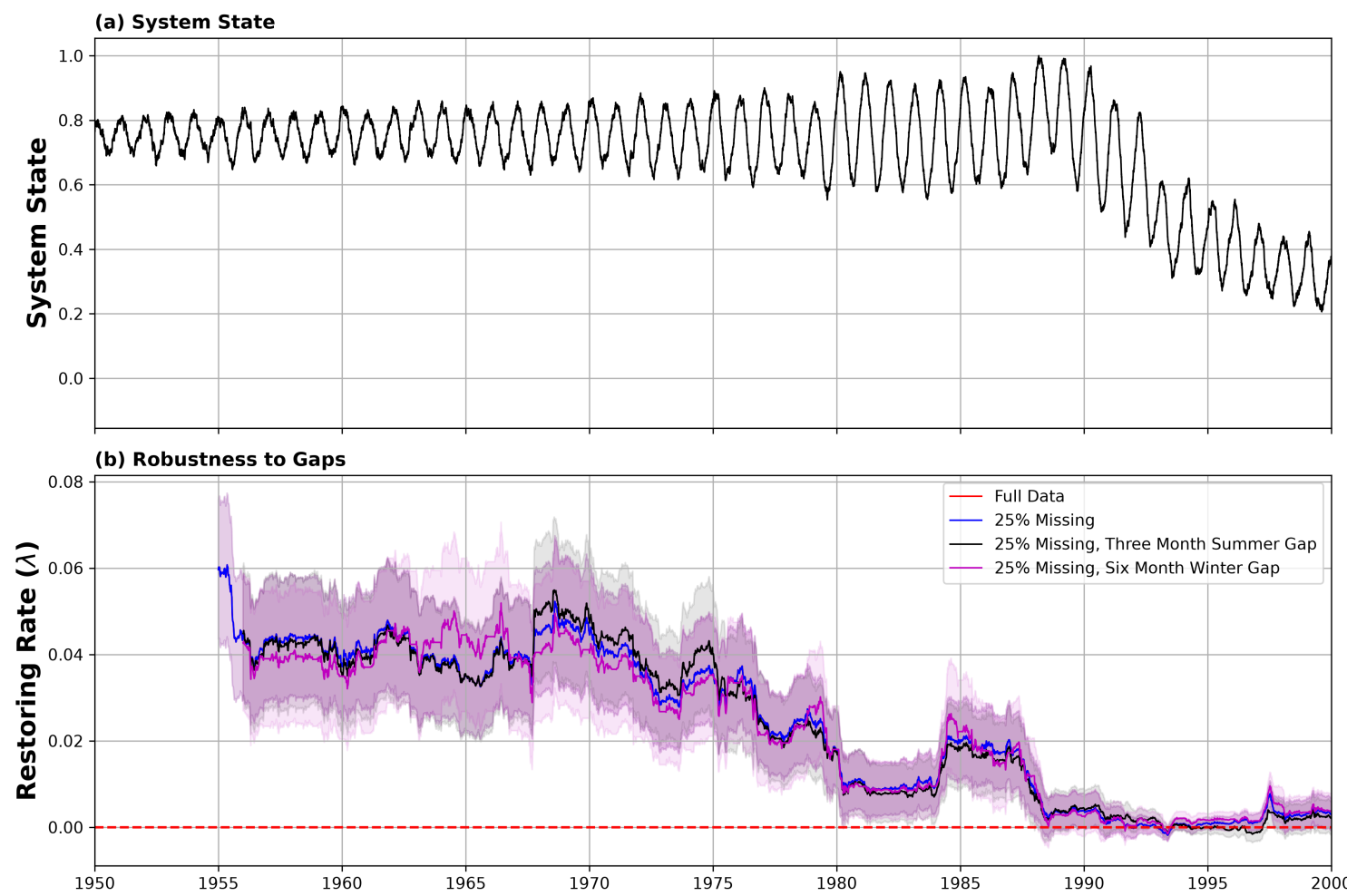

Supplemental Figure S1: **Gap Robustness.** (A) Pitchfork bifurcation model with seasonality. (B) Restoring rate λ estimated on time series realizations with variable gap percentages and gap lengths.

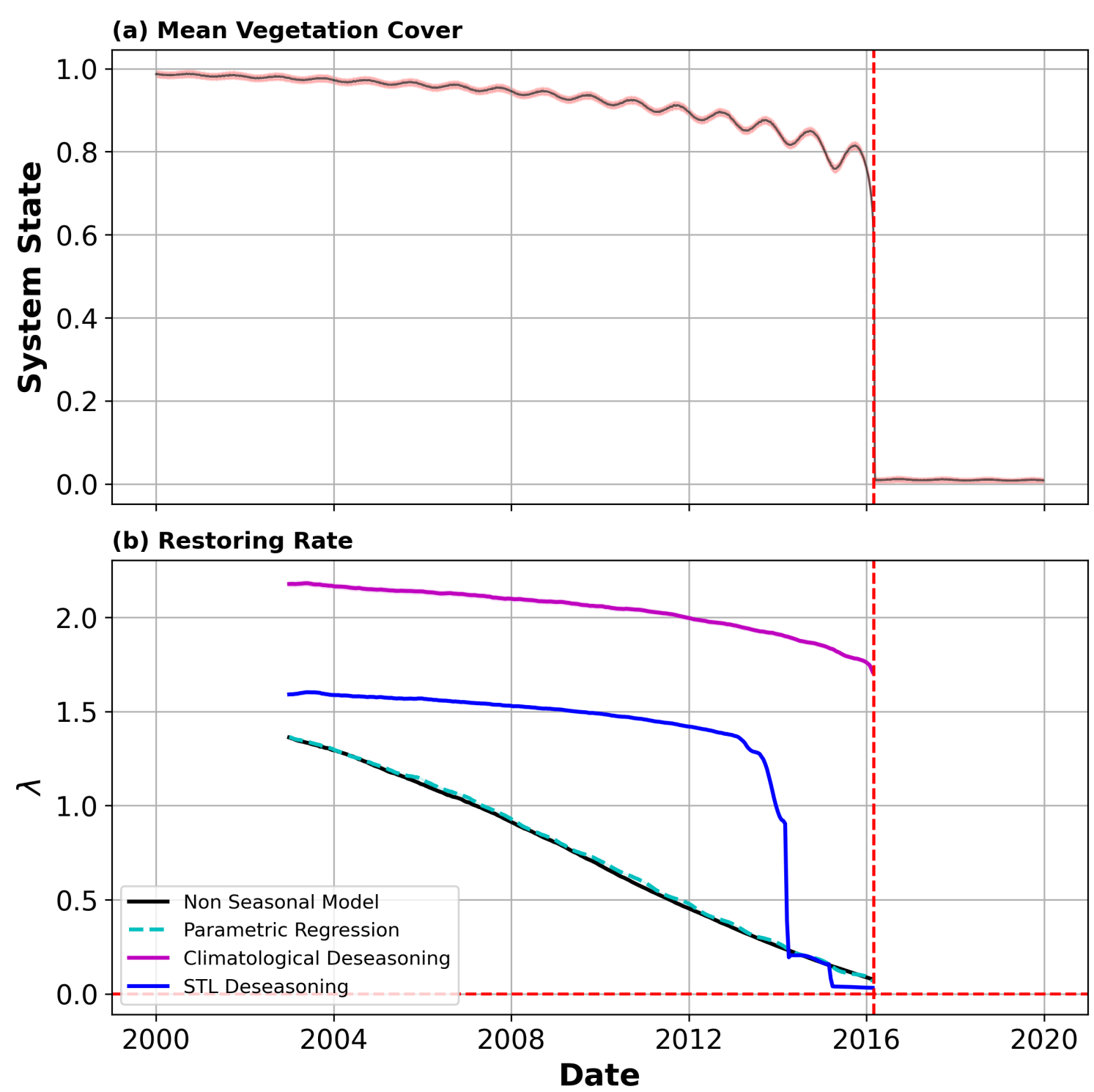

Supplemental Figure S2: **Stability of a spatially-extended vegetation model.** (A) Mean vegetation state. (B) Restoring rate λ estimated on four different data processing schemes. Note that the non-seasonal control run and parametric regression with harmonic terms (Methods) yield almost identical results.

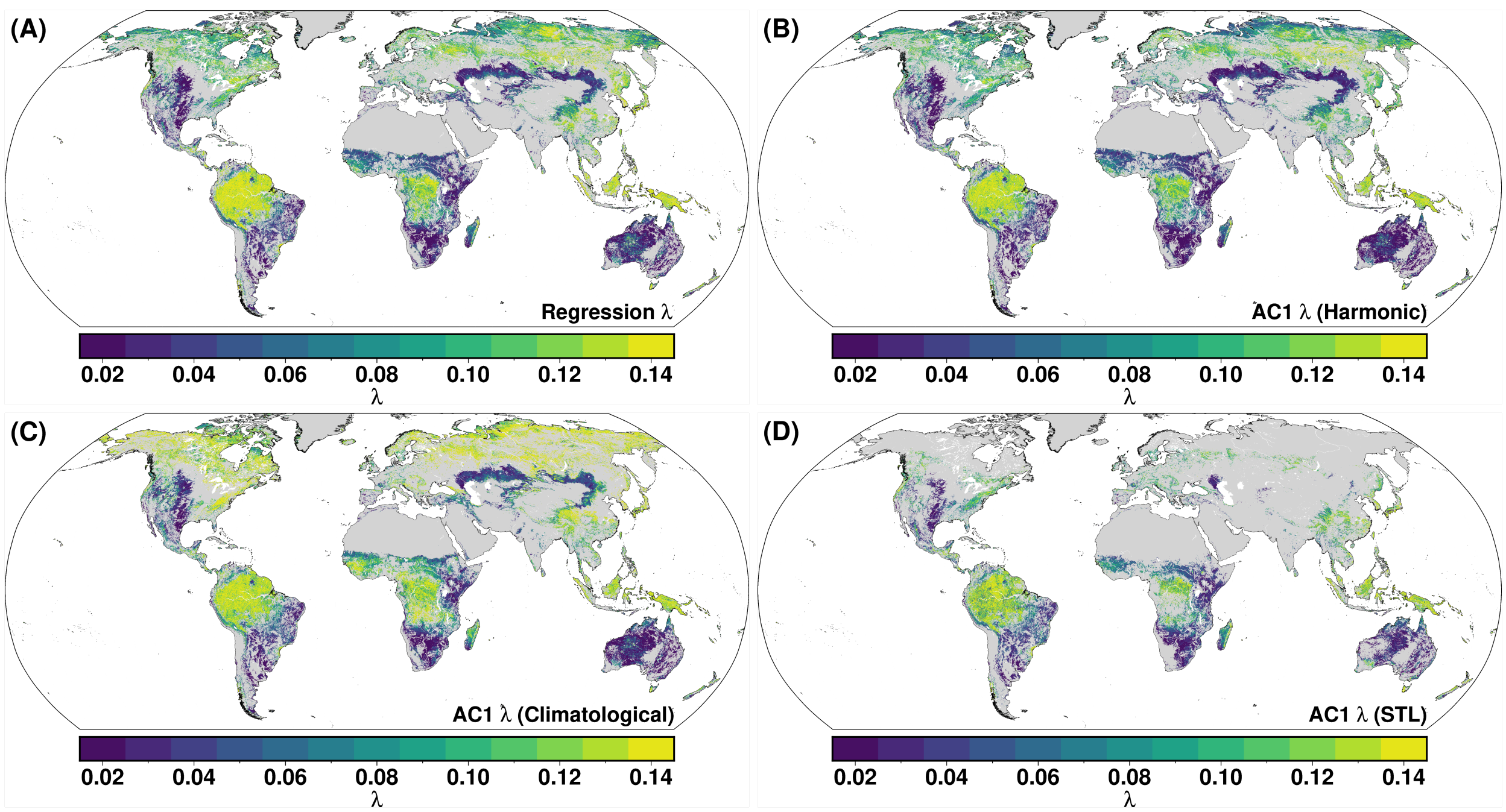

Supplemental Figure S3: **Global Vegetation Data.** (A) Regression-based λ using a harmonic-linear design matrix. (B,C,D) λ_{AC1} estimated on deseasoned and detrended data: (B) harmonic deseasoning and rolling mean detrending, (C) climatological deseasoning and rolling mean detrending, (D) STL deseasoning and detrending.

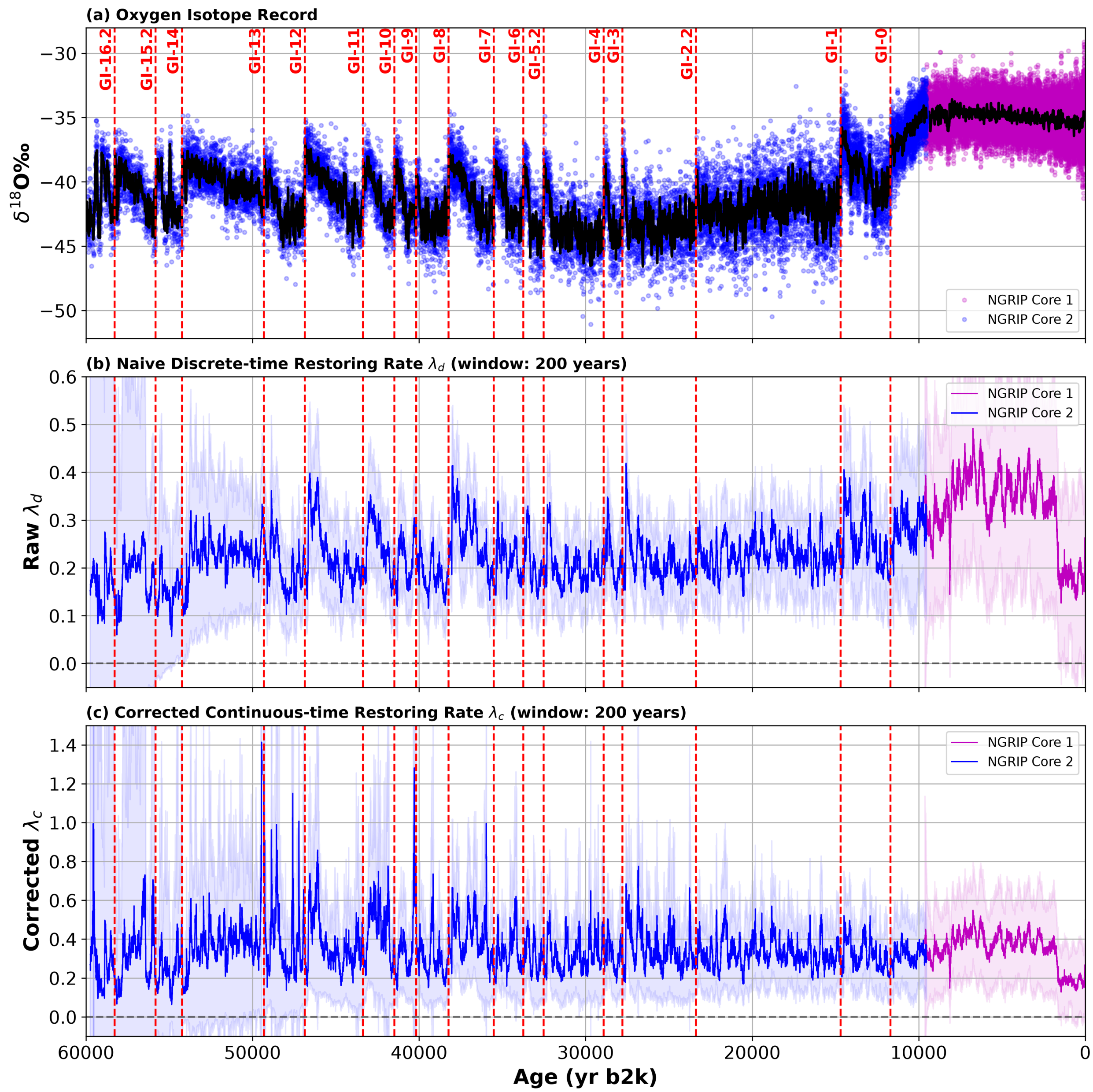

Supplemental Figure S4: **NGRIP Ice Core Data.** (A) δ^{18}O isotope records from NGRIP. Vertical lines mark DO event timing after Boers (2018). (B) Changes in discrete-time λ_d calculated on the raw time series without pre-processing, incorporating measurement and age-model uncertainties via weighted least squares (Methods). (C) Corrected λ_c accounting for changes in sampling intervals through time (Methods), showing less pronounced decreases in λ before many DO events. Shaded bounds (B,C) cover one standard deviation uncertainty in λ (Methods).

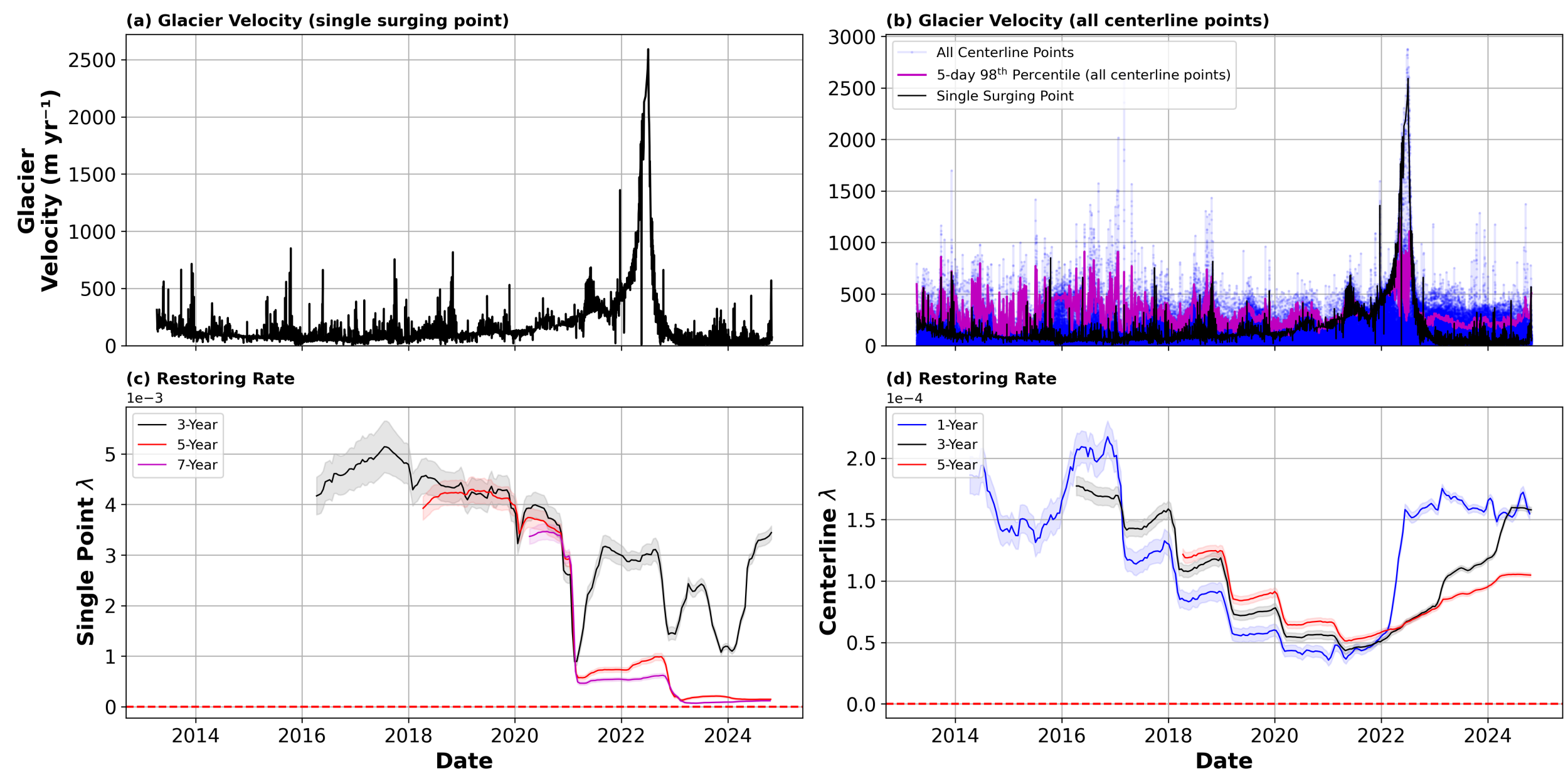

Supplemental Figure S5: **Stability of a surging glacier.** (A) Glacier velocity sampled at a single surging point and (B) over all glacier centerline points in 500 m steps. (C,D) Restoring rate λ computed for multiple moving window sizes, showing a rapid decline in stability before the onset of a major surge event. λ estimated using a parametric regression that includes a harmonic and quadratic term. Shaded bounds (C,D) cover one standard deviation uncertainty in λ.

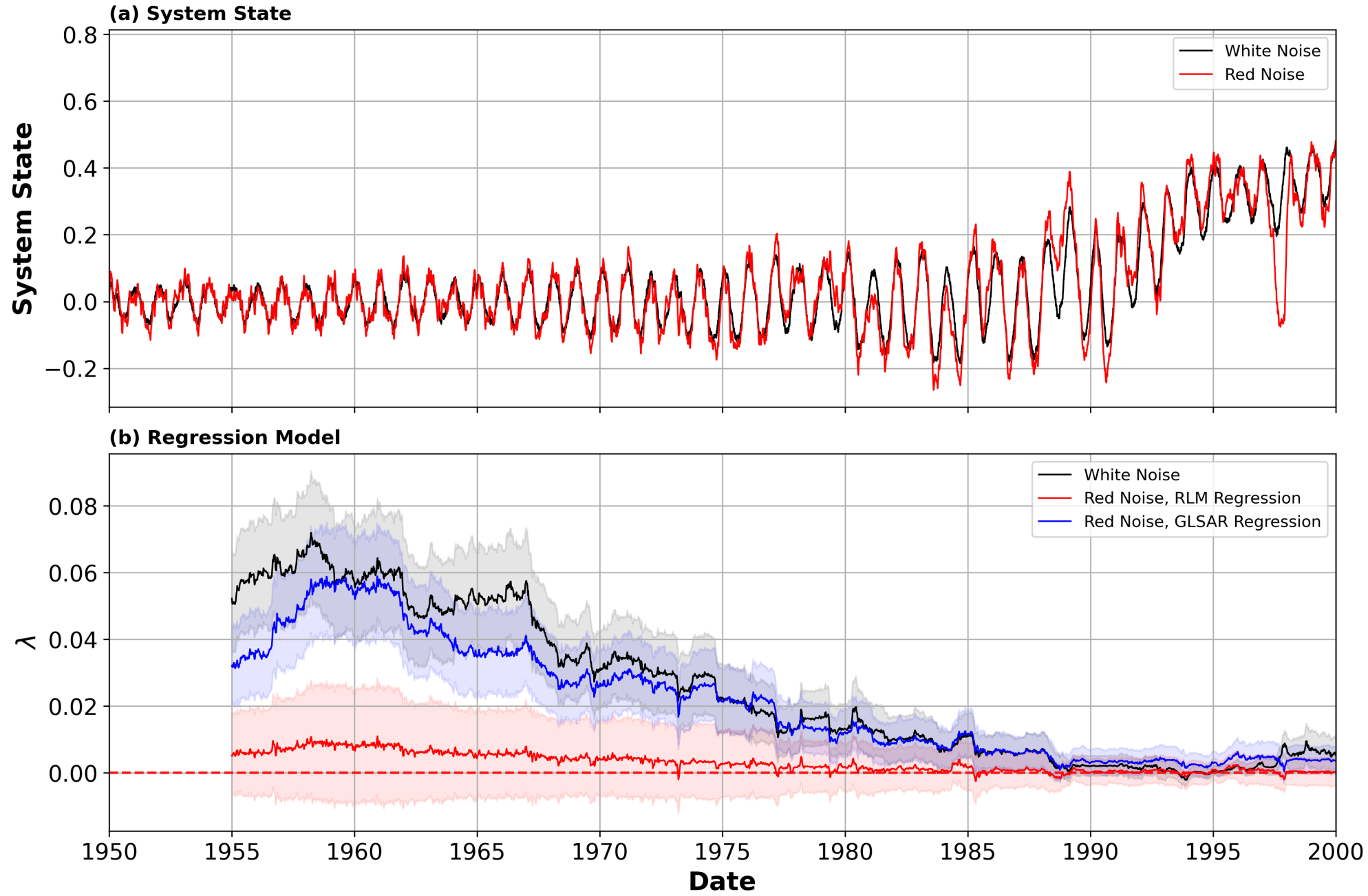

Supplemental Figure S6: **Accounting for Red Noise.** (A) Pitchfork bifurcation model with seasonality, forced by white or red noise. (B) Comparison of robust regression and GLSAR for a synthetic system with red noise forcing. Replacing the regression framework successfully accounts for the difficult noise regime.

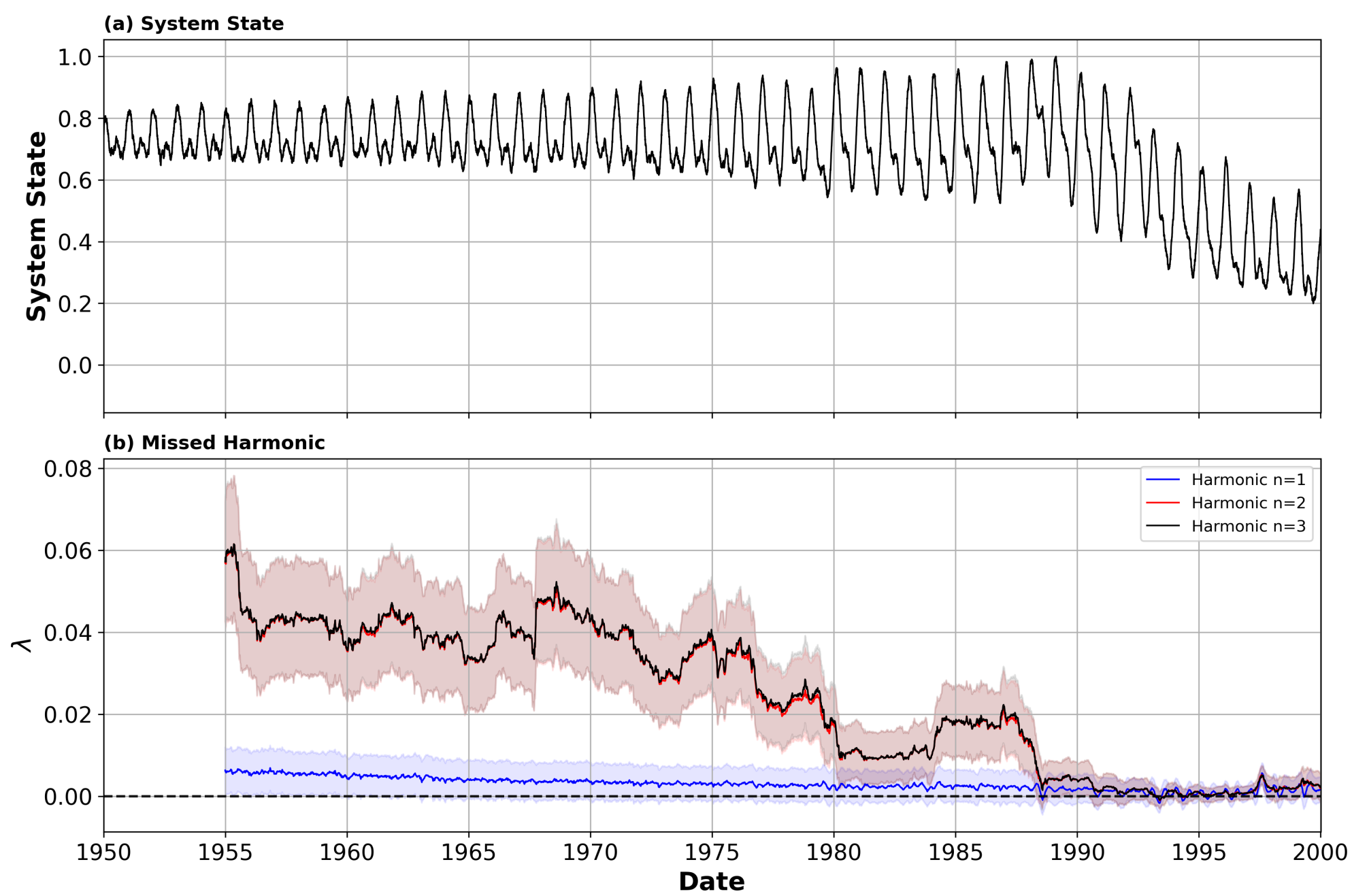

Supplemental Figure S7: **Influence of Missing Regression Factors.** (A) Pitchfork bifurcation model with annual and semi-annual harmonics. (B) Comparison of λ estimates for when too few or too many harmonic terms are included in the parametric regression.

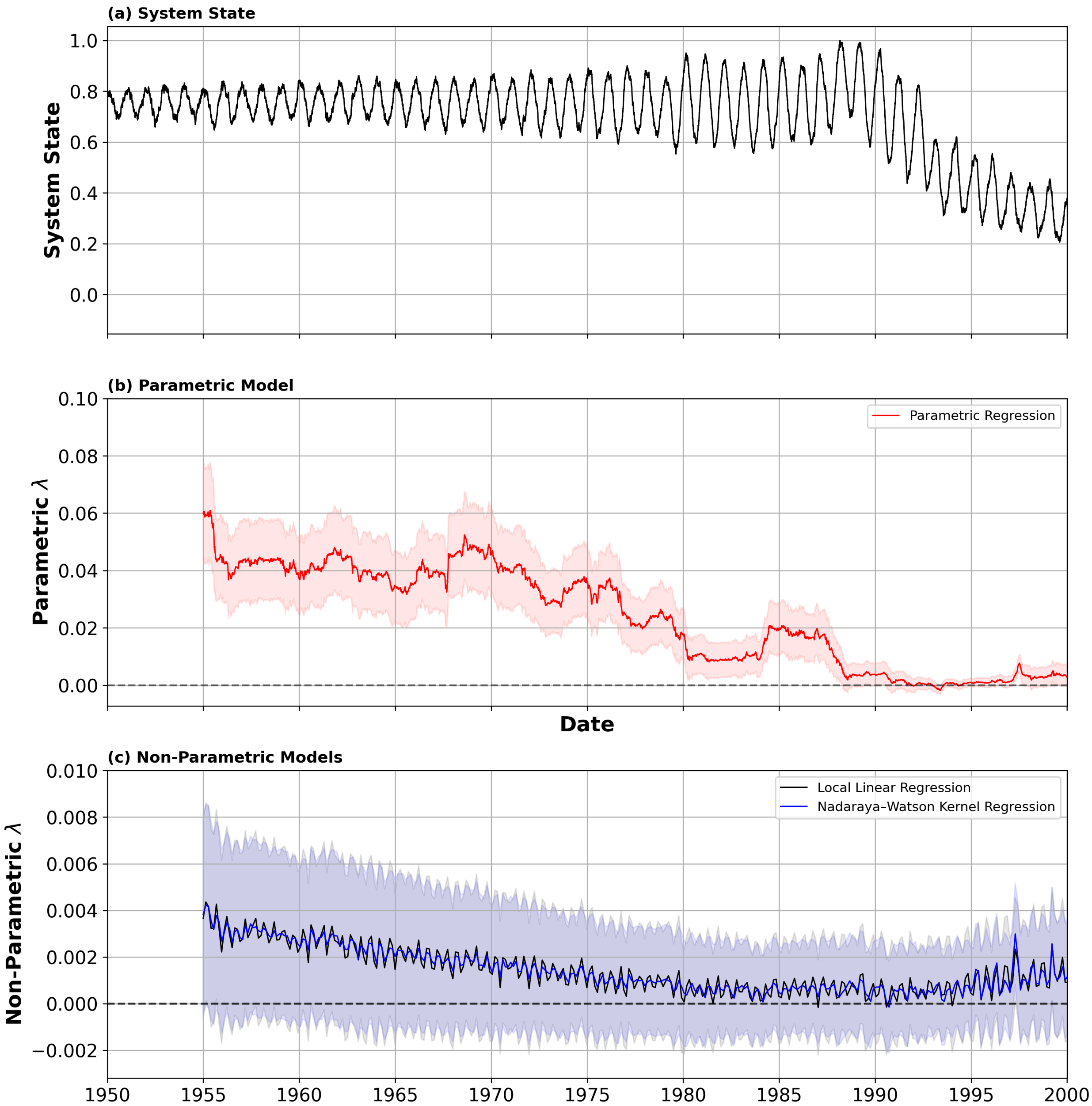

Supplemental Figure S8: **Non-Parametric Regression.** (A) Pitchfork bifurcation model with seasonality. Comparison of λ estimates for (B) parametric and (C) non-parametric approaches.

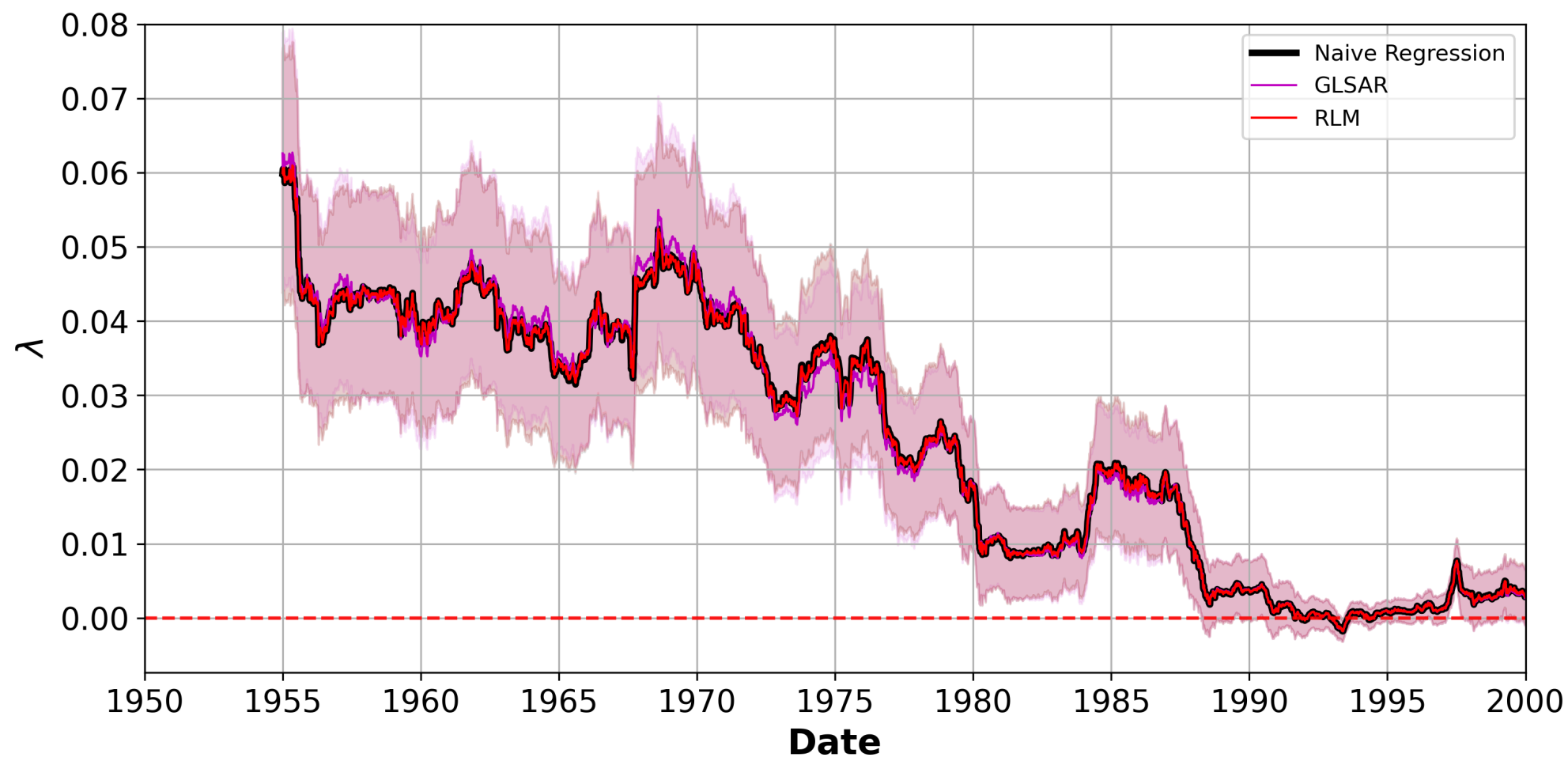

Supplemental Figure S9: **Comparison of different regression models used to recover λ.** Naive ordinary least squares (black), generalized least squares (purple) and robust regression (red) on the same pitchfork bifurcation model used in Figure 1. Depending on the system under study, some regression approaches may be more or less appropriate; our method allows for a flexible choice of regression framework.

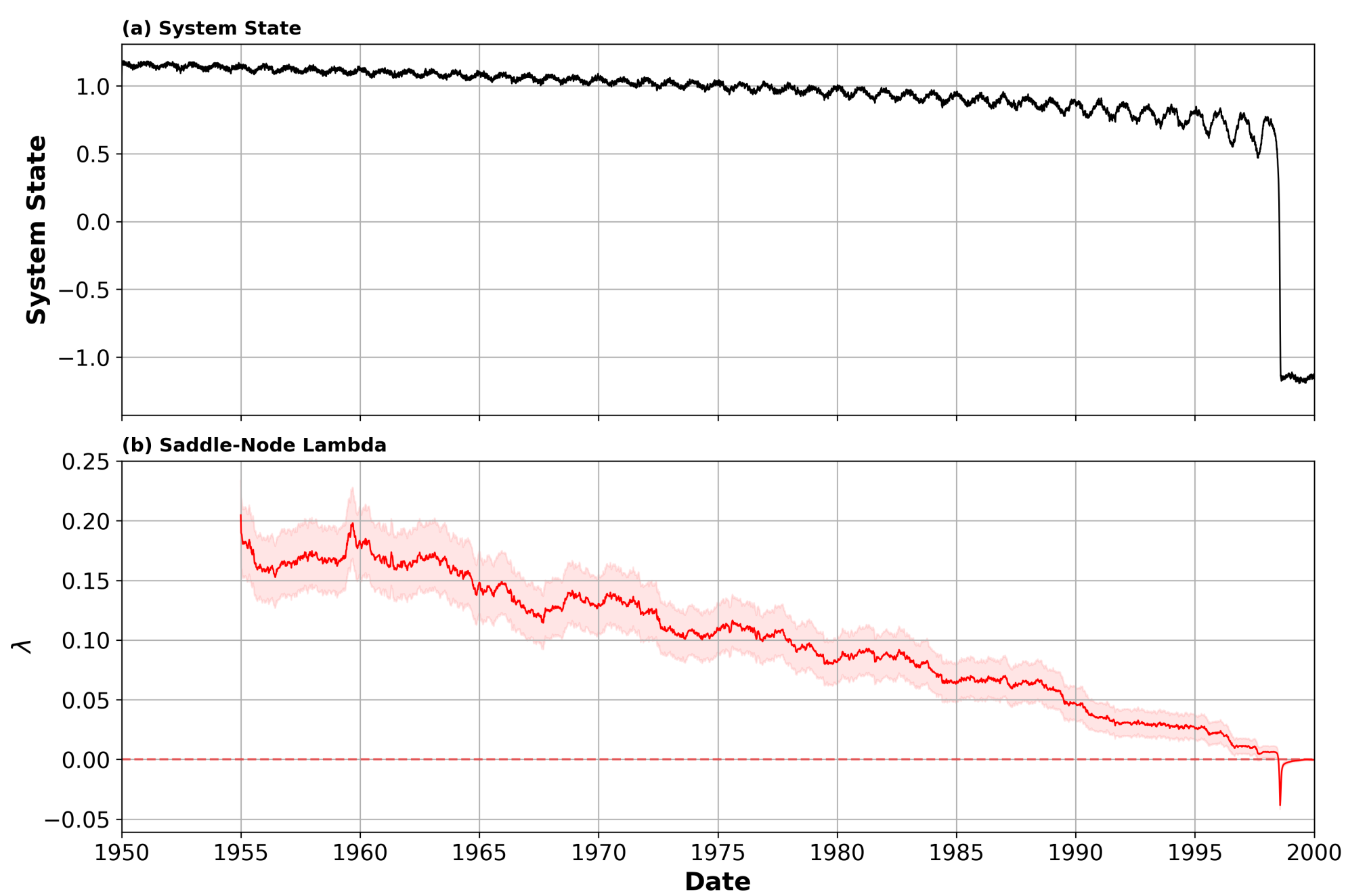

Supplemental Figure S10: **Saddle-Node Bifurcation.** (A) System state. (B) Restoring rate λ estimated using parametric regression with harmonic terms.

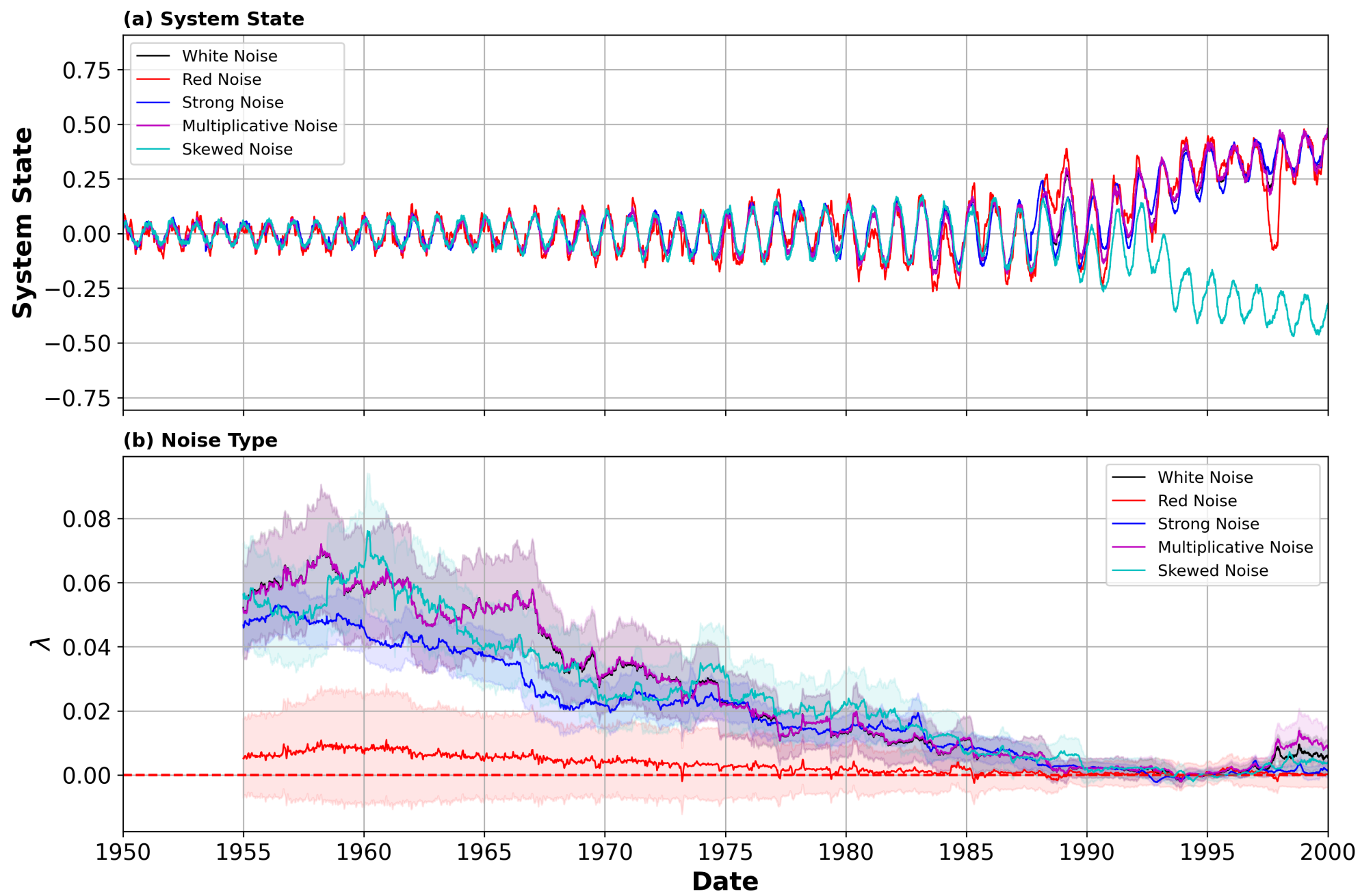

Supplemental Figure S11: **Performance of λ for different noise forcing regimes.** (A) Pitchfork bifurcation model with seasonality, forced by varying noise types. (B) Comparison of λ estimates for different noise forcing regimes. All regimes except red noise yield similar λ estimates.

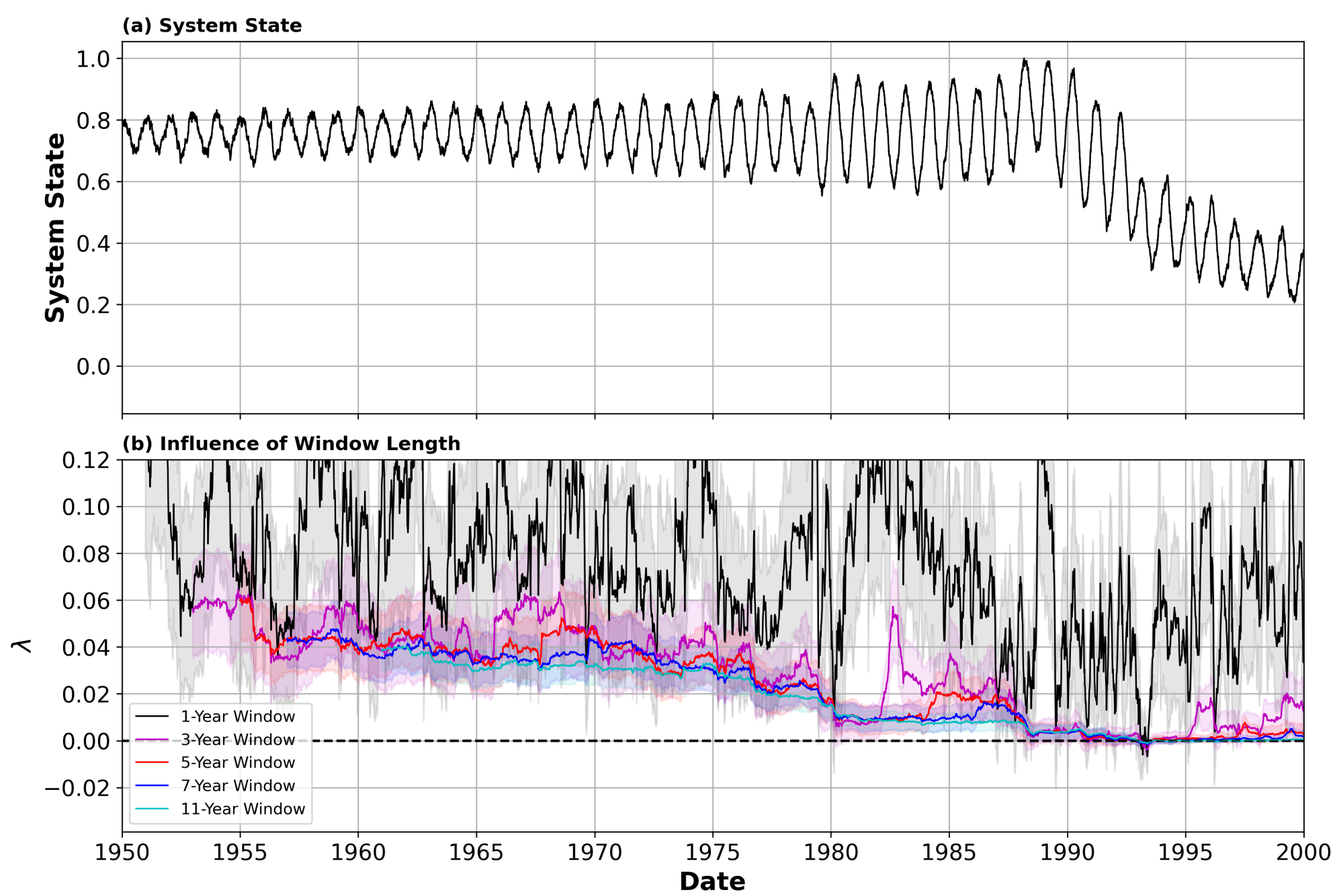

Supplemental Figure S12: **Influence of Moving Window Size.** (A) Pitchfork bifurcation model with seasonality. (B) Comparison of λ estimates for different moving window sizes. Longer moving windows generally smooth the λ estimates.

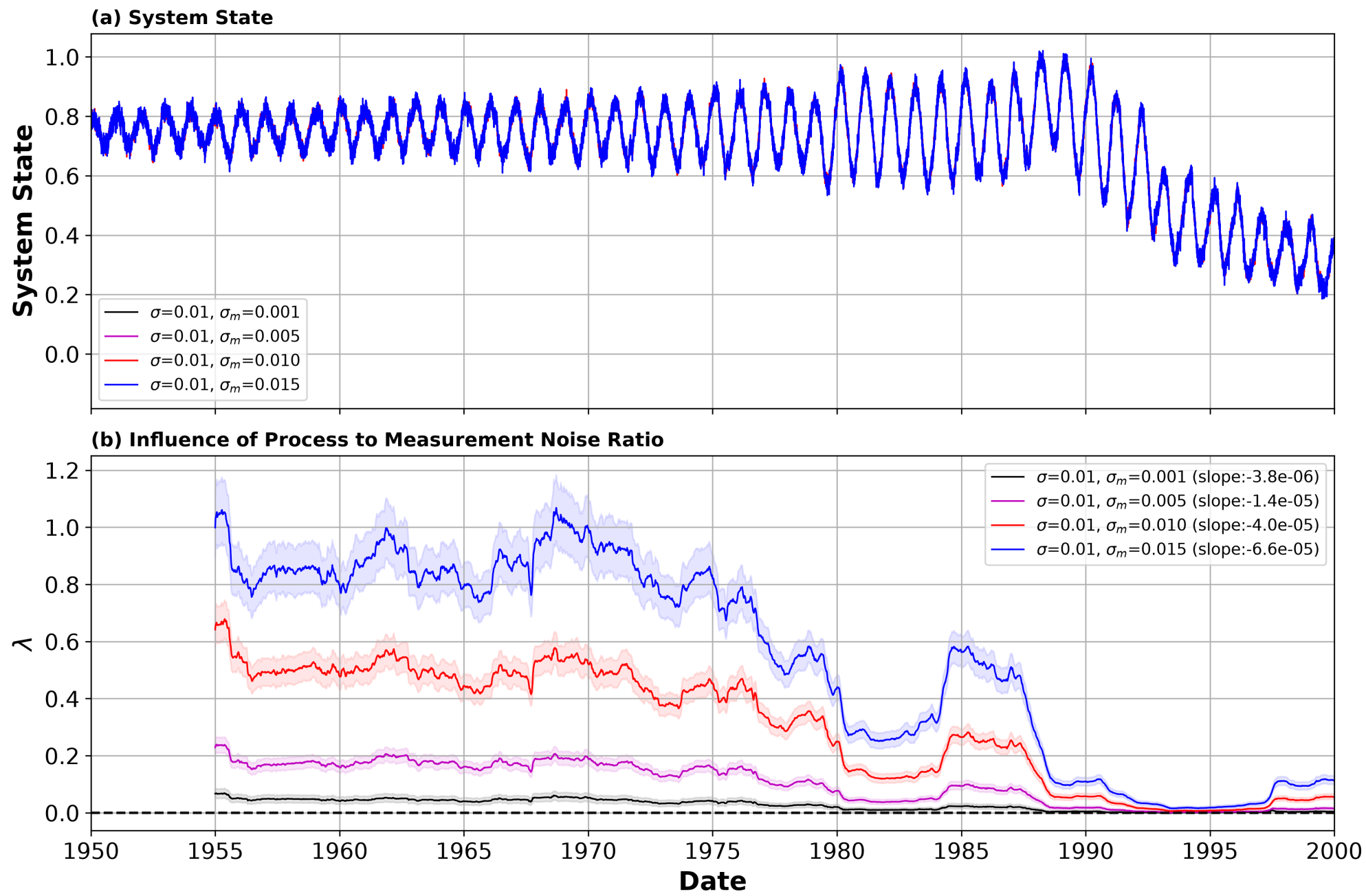

Supplemental Figure S13: **Influence of Measurement Noise.** (A) Pitchfork bifurcation model with seasonality, with varying ratios of process to measurement noise. (B) Comparison of λ estimates for different ratios of process to measurement noise. Measurement noise biases λ upwards without changing how λ evolves through time.

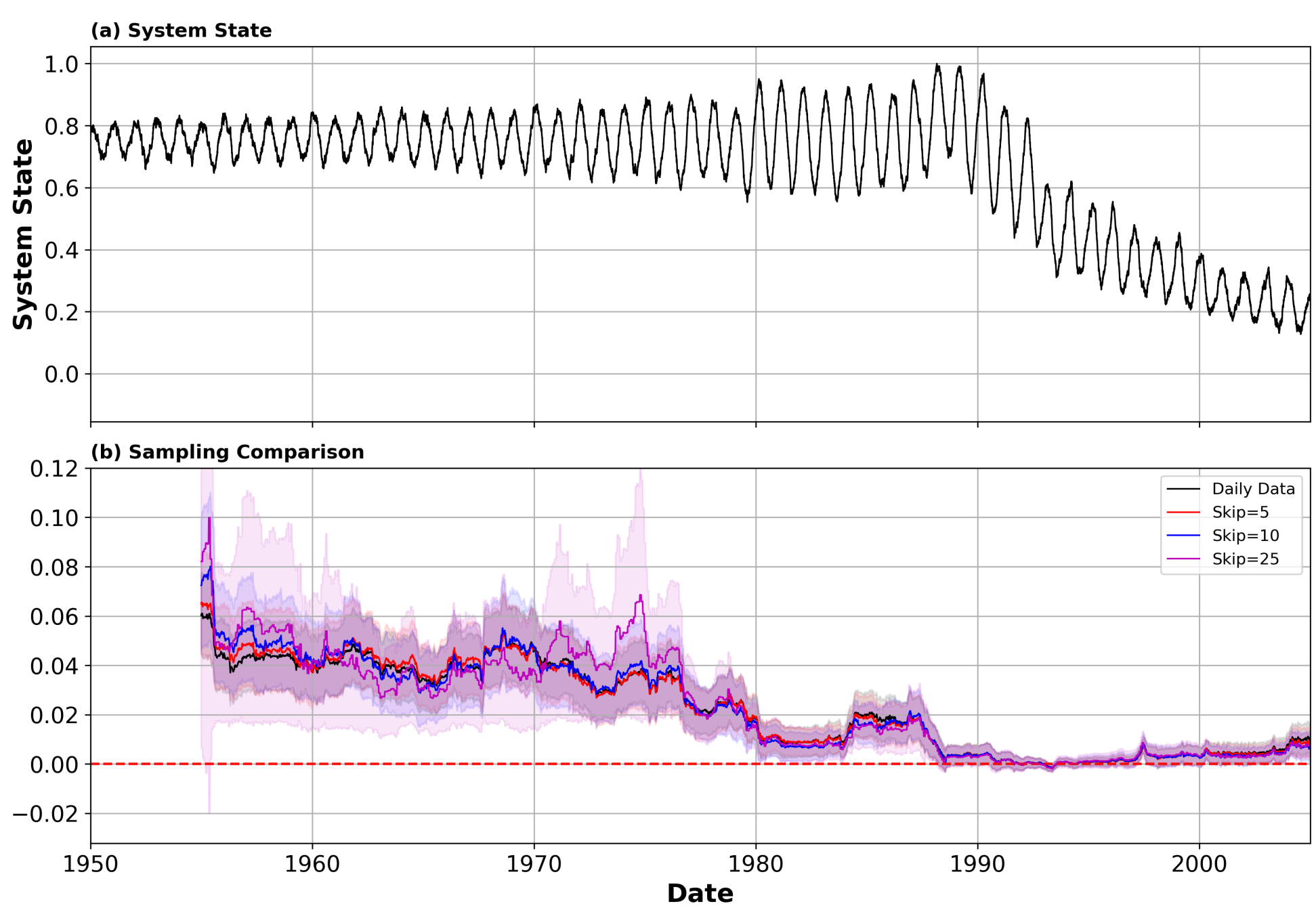

Supplemental Figure S14: **Influence of Subsampling.** (A) Pitchfork bifurcation model with seasonality. (B) Comparison of λ estimates for different temporal sub-sampling. Larger time gaps allow more stochastic noise to accumulate; we find, however, qualitatively similar λ estimates for reasonable temporal subsampling.
