# Supplementary material for: Estimating the resilience of non-stationary systems
Source: Nat Commun. 2026 Nov 6;17:11286. doi: 10.1038/s41467-026-78489-7 (PMC13743781; doi:10.1038/s41467-026-78489-7)
Supplement: Supplementary file 2 [file 41467_2026_78489_MOESM2_ESM.pdf]

nature portfolio

Peer Review File

Estimating the Resilience of Non-Stationary Systems

Corresponding Author: Dr Taylor Smith

This file contains all reviewer reports in order by version, followed by all author rebuttals in order by version.

Attachments originally included by the reviewers as part of their assessment can be found at the end of this file.

Version 0:

Reviewer comments:

Reviewer #1

(Remarks to the Author)
Comments on "Estimating the resilience of non-stationary systems"
Smith et al

This is a thought-provoking and interesting paper that highlights the problem of estimating resilience for what the authors call non-stationary systems.
The main novelty I see is that the authors propose a procedure for estimating the linearized return rate λ using regression that allows for (a) variable step sizes and (b) certain types of seasonal and climatic variation. This is definitely a meaningful development as far as I can see, and it is good to see the uncertainties plotted routinely, and error propagation seems to be treated appropriately.
My first thought was that we ALWAYS have to deal with non-stationarity in tipping problems, as we are interested in some gradual change in background (eg temperature, CO2 forcing). Then I realised that this is not what they mean – they are interested in loss of stability of states that are not equilibria, but that, for example, have seasonal variation or trend. Hence, I feel "non-stationary" is being used in a non-standard way, and this may lead to confusion if in the title. A stationary time series is one whose pdf is independent of time: a stationary system is presumably one that produces stationary time series. Then any system that is undergoing changes in attractors with time will be non-stationary. I feel it would help the readers to understand that they are really addressing resilience in systems where the mean state varies periodically or there is a trend.
I didn't pick up why the fitted linear model is called a "design matrix"?
The authors apply their methods to local measures of Vegetation resilience from the MODIS database, to Dansgaard-Oeschger events in paleoclimate and to Glacial surges. It is fair to say that the results are not entirely conclusive, but they seem to be producing higher quality estimates for the simple periodically forced model (15).
However, in (15) I was quite confused that λ appears there (but is set to zero) – is this a mistake? My understanding is that λ is an output from the estimation, not a model parameter.
On p4 the authors state that λ is constant but then that it can change seasonally. But then equation (3) does not include any variation in λ. Which is it? I am confused. I note (3) is essentially a forward Euler integrator of a scalar SDE, which is valid when the system is one-dimensional, but is there any guarantee this will be relevant for a higher-dimensional system where there will be not just one but potentially many λ? In particular, Euler integrators can behave quite badly for large timesteps, especially in highly stable systems, and so I think there will be quite stringent limits on how large the timestep can be and (3) still to be valid.
On p5 the authors claim that (3) can be reformulated for simpler (eg linear drift) components. I would like to understand this more clearly: in my mind, this is potentially not simple at all – periodically forced systems can be studied using Fourier series to approximate the response, while linear drift of an input can lead to complex nonlinear drift of solutions that will typically involve unboundedness.
One thing that I feel a bit uneasy about: the assumption of lack of seasonal variation of λ as typically I believe this will be the case, and then the overall stability is an integrated version (Floquet exponent). For example, what happens in (15) if λ is assumed to have periodic variation – does the procedure correctly estimate the average value of λ? Surely, rapid variation of λ may lead to tipping within a seasonal cycle even if the average value indicates stability? Do the authors have suggestions on how to generalise their method to allow for seasonal variability?
The authors discuss the estimation of λ quite extensively in the Methods, but the detrending and deseasoning choices are only briefly discussed. It was not clear to me how this would be generalized to other cases, and it seemed to be more art than science.

(Remarks on code availability)
I tested some of the code and this seemed to be well-written and useful.

Reviewer #2

(Remarks to the Author)
This manuscript presents an invaluable improvement to current critical slowing down (CSD) theory. Most CSD indicators used on observational datasets at present assume system stationarity and even sampling. In reality this is not the case for most applications in climate. The proposed regression method in this manuscript allows for inclusion of nonstationarity which removes the need for certain data preprocessing methods which themselves can introduce errors in the true CSD metric. I feel this contribution can be quite influential, but the manuscript needs some work before ready for publication.

General comments:

1. It is not explained why the authors use a conceptual pitchfork model as their synthetic tipping data. Classic CSD will utilise a saddle-node normal form - why was this not adopted here? Pitchfork bifurcations can be either subcritical or supercritical, with only the subcritical case I would consider a true "critical" transition in the nature of the system response. However, the stable branch then is by nature stationary so there is no drift in the equilibrium. I think the authors should motivate more why this formulation is used, which criticality of bifurcation is considered, and how these results translate to the other topologically equivalent bifurcations. A further question is whether or not one wants to identify CSD in non-"critical" transitions such as transcritical or supercritical pitchfork bifurcations, but perhaps this is out of the scope of the presented study.

2. In the main text I found confusion as to when lambda was being discussed as discrete or continuous. This is clearly presented in the Methods section. I recommend using different notation in the main text as well for clarity.

3. The discussion around Figure 1 (and Supp Figure S1) is quite confusing. How were the gaps in the data implemented? Were they randomly distributed with different sizes? How then were the "long" gaps implemented in the Supp Figure? Were these yearly and alongside additional random gaps throughout the year? Finally, the seasonal and non-seasonal control models are not clear to me in Figure 1c.

4. In the Paleoclimate Proxy Data section, it is not clear how one implemented the bias correction to lambda. I initially thought this was through the O(1/n) bias mentioned in Methods related to sample size n, but then it appeared to be related to sampling intervals. Please clarify the bias correction method here.

5. Glacier surging should be briefly described in the introduction to that section. It is only mentioned a well-documented phenomenon but not what it actually is.

6. Additionally in the Glacier Surge Prediction section, it is mentioned that nonlinear terms are included in the design matrix, but I could not find an explicit formulation for this problem. I recommend explicitly writing out the form of Equation 3 used for the regression in this case which includes nonlinear terms. The only design matrix explicit to this problem was the harmonic one which the authors explicitly say omit the nonlinear terms.

Minor points:

1. The term "tipping point" is used in the introduction, but I think "tipping threshold" is more generally adopted now.

2. "Gappy" is used at times but I do not find it very scientific. Reword if not generally used in published literature.

3. Line 218: "first pre-process the data to remove, e.g., seasonality," would read better as "first pre-process the data to remove time-dependence, e.g., seasonality,"

(Remarks on code availability)
I have not run the code explicitly but I have read through it, and it is well documented and clear how one would use it for their data time series.

Reviewer #3

(Remarks to the Author)

```
\documentclass[11pt]{article}

\usepackage[a4paper,margin=1in]{geometry}
\usepackage{amsmath,amssymb}
\usepackage{enumitem}
\usepackage{setspace}
```

\setstretch{1.15}

\begin{document}

\begin{center}
{\Large \textbf{Referee Report on "Estimating the Resilience of Non-Stationary Systems" }}\\[0.3cm]

\textbf{Authors:} Taylor Smith, Andreas Morr, Christof Sch\"otz, Niklas Boers
\end{center}

\vspace{0.5cm}

\section*{}

This manuscript addresses an important limitation in the estimation of resilience and critical slowing down (CSD) indicators from observational data. Traditional approaches based on lag-1 autocorrelation (AC1), variance, and related metrics generally rely on assumptions of stationarity, regular temporal sampling, and extensive preprocessing. These assumptions are often violated in Earth system applications, where trends, seasonality, irregular sampling, and missing observations are common.

The authors employ a regression-based framework derived from the Langevin equation that explicitly incorporates a time-dependent attractor. By representing the attractor through a design matrix (e.g., harmonic and polynomial basis functions), the method simultaneously estimates both the restoring rate (resilience parameter) and the attractor dynamics. The framework naturally accommodates irregular sampling and observational uncertainties.

The methodology is demonstrated on synthetic examples and subsequently applied to three important real-world cases: global vegetation resilience, NGRIP paleoclimate records, and glacier surge prediction. The manuscript is well written, mathematically correct, and addresses a timely and important problem in resilience estimation.

The manuscript makes several notable contributions. By moving from a sequential workflow-detrending followed by resilience estimation-to a simultaneous estimation framework, the approach reduces potential preprocessing biases. Its ability to handle irregularly sampled observations directly, without the need for interpolation, is particularly valuable for paleoclimate, remote sensing, and ecological datasets. In addition, the explicit propagation of observational and age-model uncertainties enhances the interpretability of the estimated resilience. Taken together, these advances make the framework a strong candidate for becoming a standard tool for assessing resilience in non-stationary Earth system records.

\section*{Major Comments}

\subsection*{1. Sensitivity to Temporal Window Length}

The proposed method relies on moving temporal windows to estimate local resilience. Although the authors discuss selecting window sizes according to the characteristic time scales of the system, a more systematic sensitivity analysis would strengthen the paper.

Specifically, it would be valuable to quantify how estimated restoring rates vary as a function of window length. Very short windows may lead to under-constrained regressions and larger uncertainties, whereas excessively long windows may violate the quasi-stationarity assumption underlying the local approximation.

Providing such an analysis for at least one synthetic example and one real-world application would help establish practical guidelines for future users.

\subsection*{2. Assumption Regarding Measurement Noise}

In the derivation, the authors assume that process noise dominates measurement noise. While this assumption may be appropriate in many applications, it is not universally valid.

For several paleoclimate proxies and satellite-derived observations, measurement uncertainties can be substantial and may be comparable to process variability. I therefore encourage the authors to perform synthetic experiments in which the ratio between process noise and measurement noise is systematically varied.

This would clarify the regime in which the proposed estimator remains unbiased and provide guidance regarding its applicability to noisy datasets.

\subsection*{3. Attractor Model Misspecification}

The framework requires the user to specify a functional representation of the attractor dynamics through the design matrix

(e.g., Fourier components or polynomial trends).

A natural concern is whether misspecification of the attractor model introduces biases into the estimated restoring rate. For example, if the true attractor contains higher-frequency components or nonlinear structures not represented in the chosen basis, can these unmodeled components leak into the resilience estimate?

The authors should discuss this issue more explicitly and, if possible, provide a synthetic example illustrating the impact of attractor misspecification on the inferred restoring rate.

\subsection*{4. Cross-Validation via Kramers--Moyal Coefficients}

The proposed regression framework can be interpreted as a parametric estimation of the first Kramers--Moyal coefficient $D^{(1)}$, corresponding to the deterministic drift term of the underlying stochastic process.

To further validate the methodology, I recommend that the authors compare their estimates with standard non-parametric Kramers--Moyal reconstruction techniques. In particular:

\begin{itemize}
\item Estimate the first Kramers--Moyal coefficient using the kernel-based approaches on synthetic datasets.
\item Compare the local gradient of the reconstructed drift function with the restoring rates obtained from the regression framework.
\item Demonstrate consistency between the parametric and non-parametric estimates.
\end{itemize}

Such a comparison would establish a direct connection between the proposed method and the classical stochastic-process framework underlying Langevin dynamics.

\subsection*{5. Non-Parametric Benchmarking Using Kernel Regression}

The methodology relies on a predefined representation of the time-varying attractor. Although the authors demonstrate robustness for several examples, it remains unclear how sensitive the results are to the specific basis functions chosen.

As a robustness check, I suggest employing a non-parametric benchmark such as Nadaraya--Watson kernel regression or local linear regression to estimate the evolving attractor.

The resulting resilience estimates could then be compared with those obtained from the proposed harmonic or polynomial design matrices. Agreement between the two approaches would substantially strengthen confidence that the inferred resilience trends are not artifacts of the chosen functional form.

\subsection*{6. Comparison with Alternative Reconstruction Frameworks}

The global vegetation application represents a high-dimensional spatio-temporal system in which spatial interactions and network effects may play a role.

For this reason, it would be informative to compare the reported resilience trends with those obtained using an alternative dynamical reconstruction framework such as HINTS (HiNTS: Higher-Order Interactions in N-Dimensional Time Series) or related moment-based reconstruction techniques.

While a full analysis may be beyond the scope of the current manuscript, even a limited comparison on a subset of the vegetation dataset would provide an independent validation of the inferred resilience loss and demonstrate consistency across reconstruction methodologies.

\subsection*{7. Robustness to Noise Structure}

The derivation assumes Gaussian white noise forcing. However, many Earth system records exhibit temporal correlations, heavy-tailed fluctuations, or state-dependent noise.

I encourage the authors to discuss the extent to which their restoring-rate estimates are affected by:

\begin{itemize}
\item temporally correlated (red) noise,
\item non-Gaussian forcing,
\item multiplicative or state-dependent noise.
\end{itemize}

Examining the behavior of the second Kramers--Moyal coefficient $D^{(2)}$ in the real-world examples may help determine whether diffusion effects influence the reported resilience estimates.

Such analyses would significantly strengthen the generality and robustness of the proposed framework.

\section*{Minor Comment}

\begin{enumerate}[label=\arabic*.]

\item A brief discussion of computational efficiency would be useful. Although the proposed method is clearly more sophisticated than standard AC1 estimation, readers working with large spatio-temporal datasets would benefit from an indication of computational scaling and runtime.

\end{enumerate}

\section*{Recommendation}

\textbf{Recommendation: Accept after Minor Revisions.}

The requested revisions primarily concern additional validation, robustness analyses, and clarification of methodological assumptions. These suggestions are intended to further strengthen an already strong contribution rather than to address fundamental shortcomings.

\end{document}

(Remarks on code availability)

Version 1:

Reviewer comments:

Reviewer #1

(Remarks to the Author)
Review of 726490_1 revised

The authors have made a concerted effort to address the concerns raised in the first version. Unfortunately, I now have more concerns, given I understand more of what is in the manuscript.

(a) The only details of the theoretical “pitchfork” model used to explore the method seem to be in the supplementary information, where it is said: “For the pitchfork-bifurcation time series model, we use the same model setup as was used in Smith et al. (2026).” However, there is no such reference – I assume they mean equation (3) of [13] Smith et al (2025), which is now published in Nature Comms (2026). It seems they did not update this in the revision. This is quite inadequate given that this “basic model” is explored in detail in many of the figures and is a key motivating example. I think at least the equation should be given.

(b) In spite of (a), I have a growing concern about the amount of overlap with Smith et al (2026): it is not clear to me that the conclusions about the glacial surging or ecosystem tipping are much of an advance on what was found in Smith et al (2026) with other methodology. The methodology is, however, still interesting and deserves to be published.

(c) I have concerns about the relevance of this theoretical “pitchfork”. As the case considered is supercritical, in some definitions this would not be seen as a critical transition – the attractors after bifurcation are close to those before, and so I can’t see an argument that this is modelling an “abrupt change”. This is not a very challenging example given that there is no trend that needs to be removed before the bifurcation – we are fortunate to be near an unchanging equilibrium in the absence of noise and seasonal forcing. I would expect to see evidence of more than one attractor after the bifurcation, unless the noise and forcing are carefully chosen to always end up on one branch, and possible hopping between these attractors. Is there any example in the climate literature where such a pitchfork is found?

(d) I am concerned that even in the “basic model” the authors can’t seem to get good agreement between parametric and non-parametric estimations of lambda: see Figure S8, which shows a similar trend, but the timescales are different by a factor of more than ten.

(e) The captions should consistently indicate which system/data is being explored. In particular in the supplementary info.

Minor edits:

Supplementary equation (1) is described as “a saddle node bifurcation (cubic fold)” which suggests they are synonyms. They are not.

In summary, I still recommend publication, but I am less sure of the significance of this paper.

(Remarks on code availability)

Reviewer #2

(Remarks to the Author)
The authors have greatly improved this paper, particularly with the extra analyses in the Methods section to discuss the limitations of the model. The non-parametric regression is particularly important for general application of the method.

(Remarks on code availability)
I have not reviewed the updated repository but was satisfied with the original code.

Reviewer #3

(Remarks to the Author)
Reviewer Report on "Estimating the Resilience of Non-Stationary Systems"

The authors have made a commendable effort to thoroughly address my previous comments. The addition of several supplemental figures and the comprehensive sensitivity analyses regarding window length, noise structures, and model misspecification have effectively dealt with the concerns I raised. The manuscript is now significantly more robust, and the results are presented with a high degree of transparency.

I am pleased to recommend the revised version of this manuscript for publication in Nature Communications.

I have only two minor points that the authors should consider for the final version:

Markov Assumption (Mandatory): In the theoretical framing of the methodology, it is necessary to explicitly state that by employing a Langevin-type equation to model the system dynamics, the state variable x(t) is assumed to be a Markov process. This is a fundamental underlying assumption of the framework that should be clear to the reader.

Estimation of Measurement Noise (Suggestion): As a constructive suggestion for their current or future work, the authors could note that measurement noise can be specifically estimated and separated from process noise using the approach presented in Phys. Rev. Lett. 97, 090603 (2006). Referencing this could provide additional guidance to researchers applying this method to highly noisy observational datasets.

Congratulations to all authors on a very high-quality and impactful contribution to the field.

(Remarks on code availability)

Open Access This Peer Review File is licensed under a Creative Commons Attribution 4.0 International License, which permits use, sharing, adaptation, distribution and reproduction in any medium or format, as long as you give appropriate credit to the original author(s) and the source, provide a link to the Creative Commons license, and indicate if changes were made.
In cases where reviewers are anonymous, credit should be given to 'Anonymous Referee' and the source.
The images or other third party material in this Peer Review File are included in the article's Creative Commons license, unless indicated otherwise in a credit line to the material. If material is not included in the article's Creative Commons license and your intended use is not permitted by statutory regulation or exceeds the permitted use, you will need to obtain permission directly from the copyright holder.
To view a copy of this license, visit https://creativecommons.org/licenses/by/4.0/

Reply to Reviewers - NatComms - R1

Thank you again for submitting your manuscript "Estimating the Resilience of Non-Stationary Systems" to Nature Communications. We have now received reports from 3 reviewers and, after careful consideration, we have decided to invite a major revision of the manuscript.

This is a high-quality and potentially impactful study; however, the manuscript in its current form does not yet meet the standard required for publication. In particular, the proposed method would benefit from more comprehensive validation, and several aspects of the presentation and conceptual framing require clarification. In addition, the robustness of the approach is not yet sufficiently demonstrated. Without substantial revisions, we will be unlikely to send the paper back to review.

Thank you for facilitating this review and for the high quality of the critiques. In this revision, we have added substantial new analysis documenting the influence of a range of new factors on our approach. We have also extended our framework to include non-parametric regression, which broadens the scope of systems that can be analyzed with our method.

In particular, we have added nine new Supplemental Figures, as well as one new synthetic model to improve the robustness of our results. These cover analyses of moving window sizes, process noise strength and type, measurement noise magnitude, missed parameters in our regression, and an entirely new non-parametric regression approach. We have also clarified our notation and over what reference frame it is valid in the updated manuscript.

We are glad to have received such useful comments from the Reviewers, and look forward to hearing their thoughts on the changes in the manuscript.

Reviewer #1

This is a thought-provoking and interesting paper that highlights the problem of estimating resilience for what the authors call non-stationary systems. The main novelty I see is that the authors propose a procedure for estimating the linearized return rate λ using regression that allows for (a) variable step sizes and (b) certain types of seasonal and climatic variation. This is definitely a meaningful development as far as I can see, and it is good to see the uncertainties plotted routinely, and error propagation seems to be treated appropriately.

Thank you for taking the time to review our work. We have responded to each of your concerns in turn below.

My first thought was that we ALWAYS have to deal with non-stationarity in tipping problems, as we are interested in some gradual change in background (e.g., temperature, CO_2 forcing). Then I realised that this is not what they mean – they are interested in loss of stability of states that are not equilibria, but that, for example, have seasonal variation or trend. Hence, I feel "non-stationary" is being used in a non-standard way, and this may lead to confusion if in the title. A stationary time series is one whose pdf is independent of time: a stationary system is presumably one that produces stationary time series. Then any system that is undergoing changes in attractors with time will be non-stationary. I feel it would help the readers to understand that they are really addressing resilience in systems where the mean state varies periodically or there is a trend.

Thank you for this comment. We fully agree that non-stationary data is the norm when exploring systems with gradually changing forcing, such as those we study here. In our approach, however, we investigate short moving temporal windows, under which we assume that the background forcing is constant; the non-stationarity we refer to is that within a short temporal window (e.g., seasonality) which is separate from the global non-stationarity of the long-term system trajectory, i.e. nonlinear trends.

In our experience, a typical approach to estimating CSD is to first deseason/detrend data to make it 'nominally stationary', and then estimate λ via AC1 or variance over short temporal windows. In our approach, we directly work with non-stationary (e.g., seasonal, drifting) data without preprocessing. In that sense, we account for the non-stationarity within given temporal windows directly in our λ estimates, instead of first applying a pre-processing procedure and treating the resulting time series as stationary. One of the core benefits of our approach is that we model the attractor directly, and can hence handle arbitrary non-stationary data by prescribing a functional form (e.g., an annual sinusoid). This allows us to propagate uncertainty robustly, which is not typically done – most analyses first deseason/detrend and then take the resulting time series as stationary, omitting the influence of preprocessing on any resulting resilience estimates.

We have added a note in the Introduction to make it clear from the beginning that we refer to 'stationarity' within a given temporal window.

I didn't pick up why the fitted linear model is called a "design matrix"?

We call this a 'design matrix' here to make it clear that we use a set of linear factors in our regression (e.g., seasonality, trend, quadratic terms). To us, the term design matrix is general and encompasses arbitrary regression setups. To make this clearer, we have renamed our method a 'parametric regression' approach, and updated the text throughout to make this clearer.

The authors apply their methods to local measures of vegetation resilience from the MODIS database, to Dansgaard-Oeschger events in paleoclimate and to Glacial surges. It is fair to say that the results are not entirely conclusive, but they seem to be producing higher quality estimates for the simple periodically forced model (15).

However, in (15) I was quite confused that λ appears there (but is set to zero) – is this a mistake? My understanding is that λ is an output from the estimation, not a model parameter.

We apologize for the confusion here – the λ in equation (15) refers to a linear damping coefficient (and is actually set to zero in all model runs) which was used to test moving the bifurcation point around in time during the simulation. It should have been removed from equation (15) (or renamed) for clarity – we have updated this in the revised MS.

On p4 the authors state that λ is constant but then that it can change seasonally. But then equation (3) does not include any variation in λ. Which is it? I am confused. I note (3) is essentially a forward Euler integrator of a scalar SDE, which is valid when the system is one-dimensional, but is there any guarantee this will be relevant for a higher-dimensional system where there will be not just one but potentially many λ? In particular, Euler integrators can behave quite badly for large timesteps, especially in highly stable systems, and so I think there will be quite stringent limits on how large the timestep can be and (3) still to be valid.

We refer to a constant λ within a given temporal window – if that window is sub-annual, then we would capture a sub-annual λ. We have clarified the text to make this more apparent.

The question of time validity is interesting – as time steps get longer, the amount of dynamical noise that accumulates between measurements also increases, and nonlinear behaviour in the deterministic dynamics may diverge from the time linear approach. The size of the "minimal functional" time step is hard to determine, and would be somewhat system dependent (and in the real world is often simply the time step of the data available). For the regression to be valid and stable, the time step should be substantially smaller than the system's relaxation time (i.e., $\Delta t \ll -1/\lambda$). We can test this by adding a 'skip' to the synthetic data (e.g., only using every 5th point, 10th point, etc) (Figure 1).

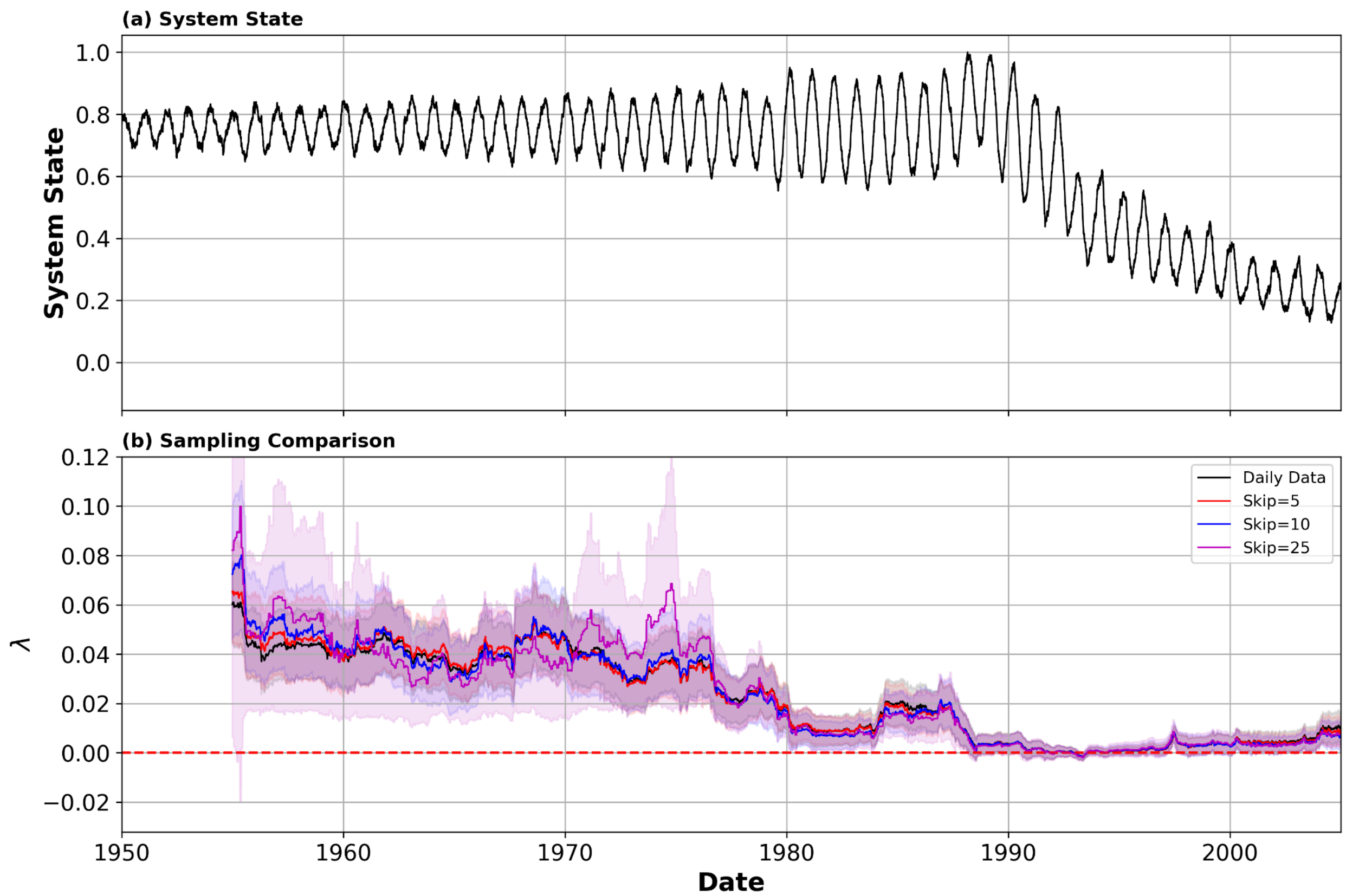

Figure 1: Comparison of sampling rates by introducing a regular skip factor into the daily data. While the recovered λ's are not identical, they are generally robust to even relatively large skips.

For reasonable skips, the estimated λ does not change substantially. This relationship will of course vary depending on the system under study, and finding the "minimal" data spacing is non-trivial. It will also to some degree depend on the forcing – if you need to resolve an annual seasonal cycle before removing that forcing, you need some minimal number of measurements to define that annual cycle. We have added this Figure to the Supplement and the related discussion in the Methods.

Multi-dimensional systems will indeed have different constraints, but would still be tractable within our framework by including multiple data variables. In this case, we would not capture a single λ, but rather a Jacobian composed of the interaction terms between multiple coupled variables; the dominant eigenvalue of that matrix would be a useful stability metric. The use of the Euler integrator in this case is still warranted and does not introduce additional errors, provided the time step is sufficiently small. For high-dimensional systems, however, the resulting time-step requirements could make the Euler approach infeasible in practice. A thorough exploration of higher-dimensional systems within this framework is the subject of work in preparation; we have updated the MS to include a short statement about this temporal resolution limitation in the Discussion.

On p5 the authors claim that (3) can be reformulated for simpler (e.g., linear drift) components. I would like to understand this more clearly: in my mind, this is potentially not simple at all – periodically forced systems can be studied using Fourier series to approximate the response, while linear drift of an input can lead to complex nonlinear drift of solutions that will typically involve unboundedness.

Thank you for this comment. We want to clarify here that we refer to 'simplicity' in this sense as the formulation of the regression problem, not necessarily simplicity in the behaviour of the system. We refer here explicitly to how we set up the regression – harmonic terms and linear terms can both be included via the regression design matrix. For a system without seasonality (e.g., the paleoclimate example), we do not include harmonic terms but rather only linear drift terms. The terms in the design matrix constrain the estimation of λ by partitioning out the attractor. In our framing, we assume that the system is locally linear around the attractor, while allowing for diverse attractor shapes modeled in the regression design matrix. As long as the system tracks the moving attractor, the residuals will remain bounded. We have updated the language to be clearer here.

One thing that I feel a bit uneasy about: the assumption of lack of seasonal variation of λ as typically I believe this will be the case, and then the overall stability is an integrated version (Floquet exponent). For example, what happens in (15) if λ is assumed to have periodic variation – does the procedure correctly estimate the average value of λ? Surely, rapid variation of λ may lead to tipping within a seasonal cycle even if the average value indicates stability? Do the authors have suggestions on how to generalise their method to allow for seasonal variability?

We agree that in natural systems there will generally be variability in λ throughout the year – recovery rates for vegetation, for example, would be faster during the spring than during the winter. In our examples, however, we limit ourselves to temporal windows long enough to resolve a sinusoidal curve (e.g., annual seasonality). For very short temporal windows, a sinusoid is very difficult to distinguish from a straight line; we could, in principle, resolve sub-annual λ changes via using linear terms instead of harmonic terms in sub-annual temporal windows, but would run into data limitations for many real-world examples. We hence use temporal windows that span at least one full year in order to partition seasonality and λ successfully, and hence cannot resolve sub-annual λ changes when there is substantial seasonality.

With enough data, one could estimate λ with our method over shorter time windows, but correctly partitioning out λ, seasonality, and any other nonlinear background trend becomes harder as the number of data samples shrinks. Some other approaches, such as those which rely on spatial statistics, could be used to estimate changes in λ over shorter time windows, but those methods come with their own drawbacks.

For seasonal data, we recommend at least one full year of data as the temporal window in order to separate seasonality from λ. Hence, our λ estimates are effectively annual averages computed over a full annual period or the length of the moving temporal window.

The authors discuss the estimation of λ quite extensively in the Methods, but the detrending and deseasoning choices are only briefly discussed. It was not clear to me how this would be generalized to other cases, and it seemed to be more art than science.

To avoid any misunderstanding, we only use deseasoning/detrending in Figure 1 to establish a baseline comparison with our synthetic data – our method incorporates seasonality and trends via parametric regression, and hence does not require standard preprocessing. We agree that deseasoning/detrending is to some degree an art – there is no one perfect method, and the choice can leave strong traces in the inferred λ estimates via, e.g., AC1. This is one of the core benefits of our method, in that we incorporate uncertainty in the attractor shape (e.g., seasonal cycle) directly into our λ estimates via a single regression, rather than first removing seasonality and then treating the resulting residuals as 'perfect' estimates of the underlying system without seasonality.

Reviewer #1 (Remarks on code availability):

I tested some of the code and this seemed to be well-written and useful.

Thank you for taking the time to review the code. We have updated the code repository to include plotting scripts for all Figures, as well as an update to the main function to include non-parametric regression.

Reviewer #2:

This manuscript presents an invaluable improvement to current critical slowing down (CSD) theory. Most CSD indicators used on observational datasets at present assume system stationarity and even sampling. In reality this is not the case for most applications in climate. The proposed regression method in this manuscript allows for inclusion of nonstationarity which removes the need for certain data preprocessing methods which themselves can introduce errors in the true CSD metric. I feel this contribution can be quite influential, but the manuscript needs some work before it is ready for publication.

Thank you for your positive review of our work. We have answered your concerns in-line below.

General comments:

1. It is not explained why the authors use a conceptual pitchfork model as their synthetic tipping data. Classic CSD will utilise a saddle-node normal form - why was this not adopted here? Pitchfork bifurcations can be either subcritical or supercritical, with only the subcritical case I would consider a true "critical" transition in the nature of the system response. However, the stable branch then is by nature stationary so there is no drift in the equilibrium. I think the authors should motivate more why this formulation is used, which criticality of bifurcation is considered, and how these results translate to the other topologically equivalent bifurcations. A further question is whether or not one wants to identify CSD in non-"critical" transitions such as transcritical or supercritical pitchfork bifurcations, but perhaps this is out of the scope of the presented study.

Thank you for this comment. In our framework, the type of bifurcation is not generally recoverable – we do not attempt to estimate the eigenvalues that may allow for such an identification. Our approach rather provides a general framework to anticipate abrupt transitions, which is applicable to many types of state changes.

We find qualitatively the same results from our method when implementing a simple saddle-node rather than pitchfork bifurcation (Figure 2). We chose a pitchfork model here as it is relatively less sensitive to noise-induced transitions, making it easier to test how λ changes as the system control parameter is varied. We have included this new saddle-node model and corresponding Figure in the Supplement for completeness.

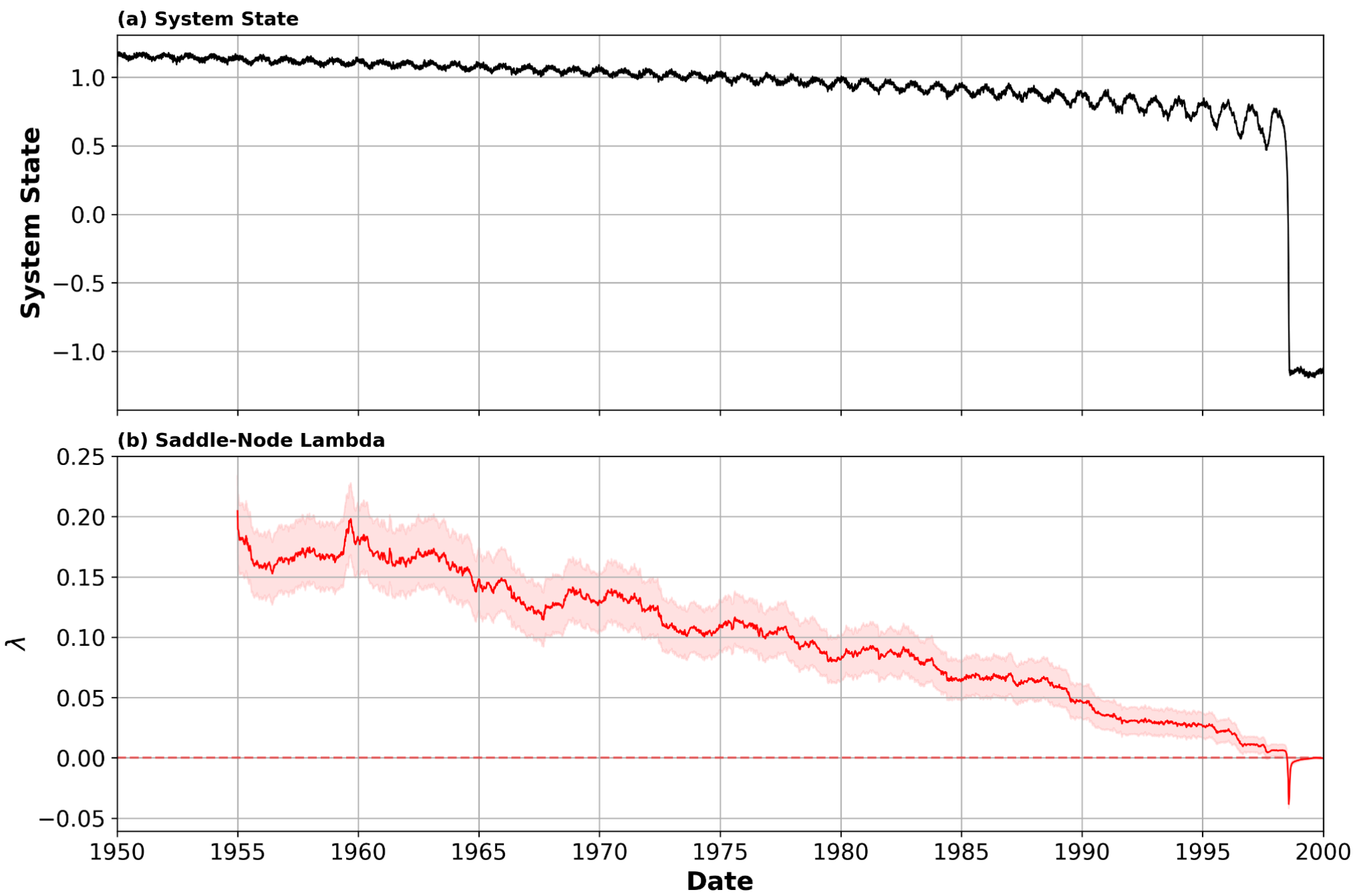

Figure 2: A saddle-node rather than pitchfork bifurcation model. The model specification can be found in the updated Supplement and the code has been added to Zenodo.

2. In the main text I found confusion as to when lambda was being discussed as discrete or continuous. This is clearly presented in the Methods section. I recommend using different notation in the main text as well for clarity.

Thank you for this comment. For legibility for a general audience, we have added a note in p6 when the discrete-vs-continuous time λ distinction is first made, and specified that our Figures always use the continuous-time (corrected) λ unless otherwise noted. We hesitate to specify λ_d and λ_c throughout, given that in many cases we refer more generally to λ, and do not want to introduce three terms for the same idea unless strictly necessary (e.g., as it is in the Methods section).

3. The discussion around Figure 1 (and Supp Figure S1) is quite confusing. How were the gaps in the data implemented? Were they randomly distributed with different sizes? How then were the "long" gaps implemented in the Supp Figure? Were these yearly and alongside additional random gaps throughout the year? Finally, the seasonal and non-seasonal control models are not clear to me in Figure 1c.

The short gaps were implemented through a random sampling approach – we chose some percentage of the time series and removed a randomly selected set of data points comprising that percentage. For the longer gaps, we focused them on specific months of the year and removed entire months of data at once. The long-gap data also had random short gaps removed. We have added this time series degradation function to Zenodo, expanded the

description in the Methods, and added further text describing these gaps in the discussion of Figure 1.

The seasonal and non-seasonal models are the output of running the model with some periodic amplitude and the same model with an amplitude of zero. In this case, given the same random seed, starting value, and forcing, we treat the model with a seasonal amplitude of zero as a non-seasonal surrogate for the seasonal model in order to test how well we handle seasonality in our data. We have clarified this point in the updated MS.

4. In the Paleoclimate Proxy Data section, it is not clear how one implemented the bias correction to lambda. I initially thought this was through the O(1/n) bias mentioned in Methods related to sample size n, but then it appeared to be related to sampling intervals. Please clarify the bias correction method here.

Thank you for pointing this out – there are indeed two related corrections here that overlap. On one hand, we correct for variable sampling densities by converting from discrete-time to continuous-time λ. This is much more important for paleoclimate data, which can have large gaps between samples, than for systems like vegetation with relatively small temporal gaps. On top of that correction, we expand the uncertainty envelope for paleoclimate data using the mean counting error (MCE) provided with the ice core data, which can be considered an independent source of measurement uncertainty (i.e., there is uncertainty in the isotope values from the machine measurement technique, and uncertainty in the spacing of the ice layers from how they are counted). We use that uncertainty – alongside the sampling interval – to estimate timing errors, which can be directly propagated into uncertainty in dx/dt and hence λ. We do not perform an O(1/n) bias correction in the MS, as we rely on large enough samples that this correction is not needed. The O(1/n) bias is mentioned at the start of the Methods section for completeness in case our method is eventually applied to smaller samples by future researchers.

5. Glacier surging should be briefly described in the introduction to that section. It is only mentioned as a well-documented phenomenon but not what it actually is.

We have added further description to the start of the glacier section.

6. Additionally in the Glacier Surge Prediction section, it is mentioned that nonlinear terms are included in the design matrix, but I could not find an explicit formulation for this problem. I recommend explicitly writing out the form of Equation 3 used for the regression in this case which includes nonlinear terms. The only design matrix explicit to this problem was the harmonic one which the authors explicitly say omit the nonlinear terms.

We have added the explicit formula to the Methods section.

Minor points:

1. The term "tipping point" is used in the introduction, but I think "tipping threshold" is more generally adopted now.

2. "Gappy" is used at times but I do not find it very scientific. Reword if not generally used in published literature.

3. Line 218: "first pre-process the data to remove, e.g., seasonality," would read better as "first pre-process the data to remove time-dependence, e.g., seasonality,"

We have updated the MS after these suggestions.

Reviewer #2 (Remarks on code availability):

I have not run the code explicitly but I have read through it, and it is well documented and clear how one would use it for their data time series.

Thank you for taking the time to review the code. The code repository has been updated to include plotting scripts for all Figures for clarity.

Reviewer #3:

This manuscript addresses an important limitation in the estimation of resilience and critical slowing down (CSD) indicators from observational data. Traditional approaches based on lag-1 autocorrelation (AC1), variance, and related metrics generally rely on assumptions of stationarity, regular temporal sampling, and extensive preprocessing. These assumptions are often violated in Earth system applications, where trends, seasonality, irregular sampling, and missing observations are common. The authors employ a regression-based framework derived from the Langevin equation that explicitly incorporates a time-dependent attractor. By representing the attractor through a design matrix (e.g., harmonic and polynomial basis functions), the method simultaneously estimates both the restoring rate (resilience parameter) and the attractor dynamics. The framework naturally accommodates irregular sampling and observational uncertainties. The methodology is demonstrated on synthetic examples and subsequently applied to three important real-world cases: global vegetation resilience, NGRIP paleoclimate records, and glacier surge prediction. The manuscript is well written, mathematically correct, and addresses a timely and important problem in resilience estimation.

The manuscript makes several notable contributions. By moving from a sequential workflow – detrending followed by resilience estimation – to a simultaneous estimation framework, the approach reduces potential preprocessing biases. Its ability to handle irregularly sampled observations directly, without the need for interpolation, is particularly valuable for paleoclimate, remote sensing, and ecological datasets. In addition, the explicit propagation of observational and age-model uncertainties enhances the interpretability of the estimated resilience. Taken together, these advances make the framework a strong candidate for becoming a standard tool for assessing resilience in non-stationary Earth system records.

Thank you for taking the time to review the manuscript and for your detailed and constructive comments. We have addressed each of your concerns in-line below.

Major Comments

1. Sensitivity to Temporal Window Length

The proposed method relies on moving temporal windows to estimate local resilience. Although the authors discuss selecting window sizes according to the characteristic time scales of the system, a more systematic sensitivity analysis would strengthen the paper.

Specifically, it would be valuable to quantify how estimated restoring rates vary as a function of window length. Very short windows may lead to under-constrained regressions and larger uncertainties, whereas excessively long windows may violate the quasi-stationarity assumption underlying the local approximation. Providing such an analysis for at least one synthetic example and one real-world application would help establish practical guidelines for future users.

Thank you for raising this point. Window length will indeed have a control on the conciseness of λ estimates, particularly as the moving windows get close to any transition. Shorter windows will

resolve high-frequency changes faster, and longer windows will smooth out any rapid changes in stability. We limit ourselves here to a minimum of one year of data in order to properly resolve seasonal oscillations, though we note that in a simple synthetic test case, a one year moving window performs very poorly and has trouble correctly partitioning the harmonic terms out (Figure 3).

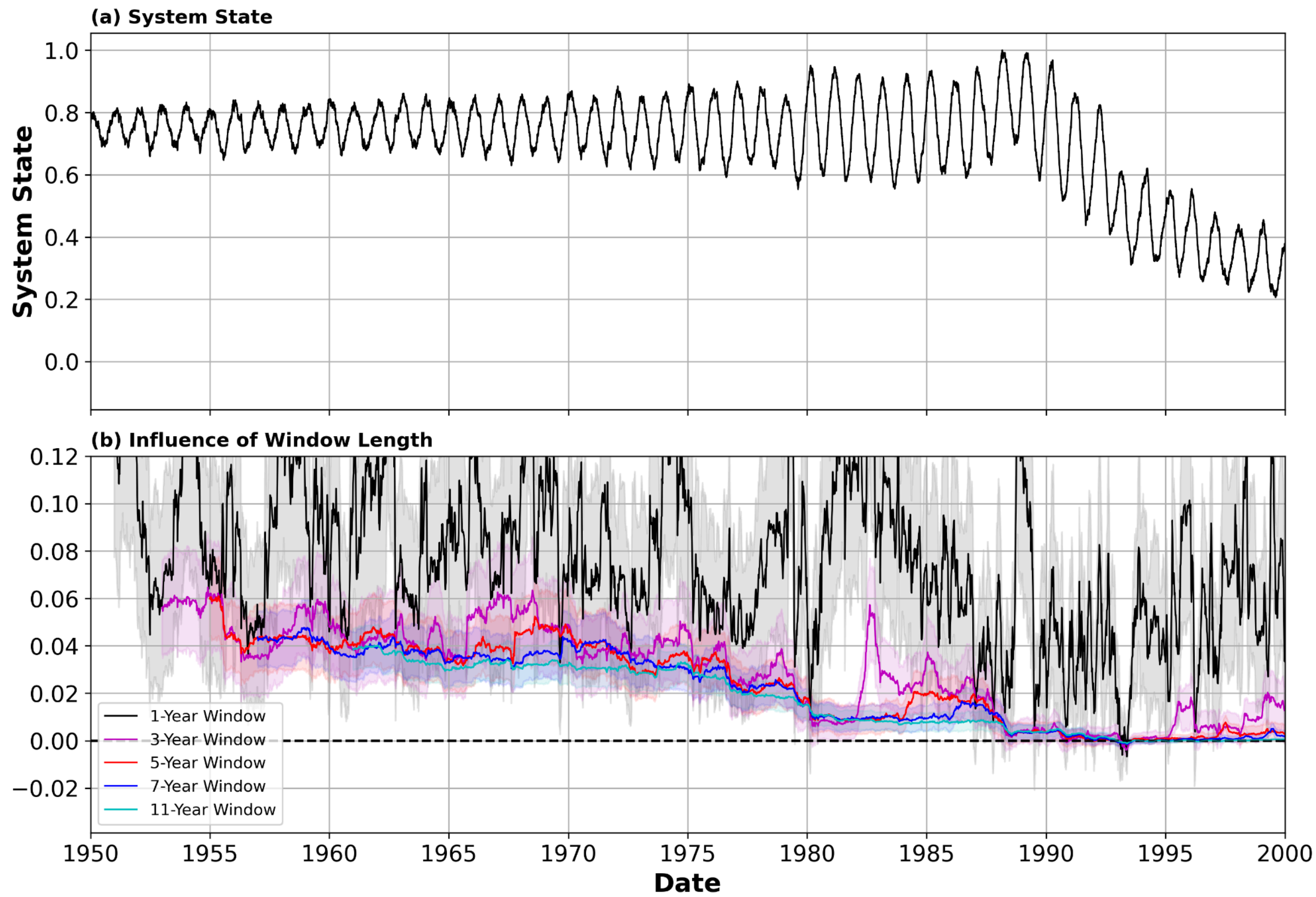

Figure 3: The influence of changing the moving window length on a synthetic time series example (cf. Figure 1).

Over longer windows, the λ estimates become increasingly smooth as the regression is better conditioned. There is no one optimal window length – if the drift towards instability happens very slowly, a long window length may work well to capture the slow movement of λ. If, on the other hand, a critical transition happens very fast, long windows will smooth over that short change, and can obscure the changes in λ of interest.

This is also the case for real-world data (Figure 4). Longer moving windows for the 1D glacier case yield smoother λ estimates, though we find the same warning signal (albeit with shorter warning as the moving windows are much longer). We can motivate shorter windows and recover qualitatively the same warning signal for the 2D glacier case, which indicates that the ‘out of phase’ nature of a collection of glacier velocity time series helps to resolve shorter temporal windows. Again, all moving windows tested recover qualitatively the same warning signal, with a different warning window.

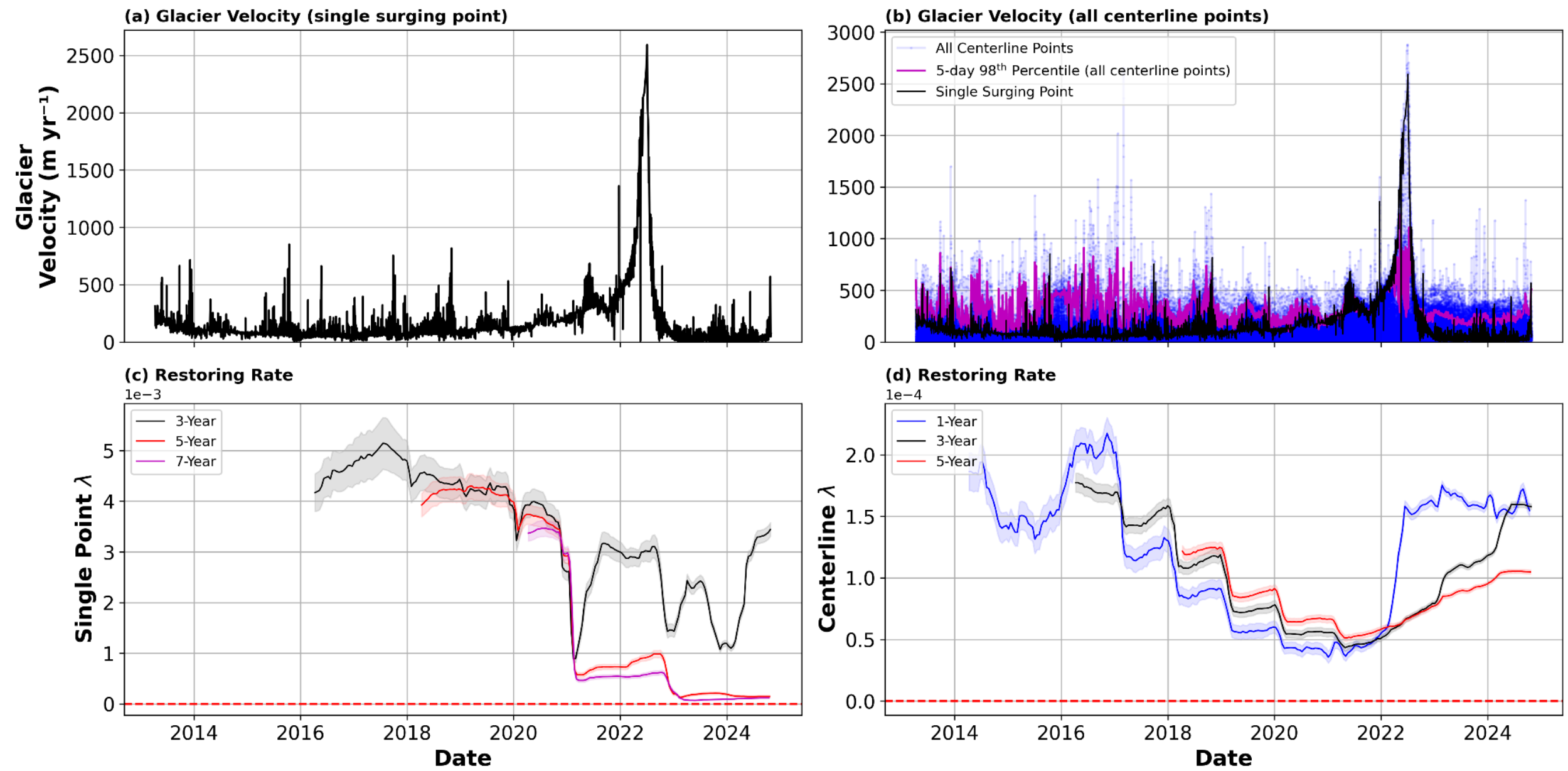

Figure 4: The influence of changing the moving window length on the glacier time series example. Longer windows generally yield smoother λ estimates, with a shorter warning before the critical transition.

We have added this discussion to the manuscript, alongside these new Supplemental Figures.

2. Assumption Regarding Measurement Noise
In the derivation, the authors assume that process noise dominates measurement noise. While this assumption may be appropriate in many applications, it is not universally valid. For several paleoclimate proxies and satellite-derived observations, measurement uncertainties can be substantial and may be comparable to process variability. I therefore encourage the authors to perform synthetic experiments in which the ratio between process noise and measurement noise is systematically varied. This would clarify the regime in which the proposed estimator remains unbiased and provide guidance regarding its applicability to noisy datasets.

Thank you for this comment. We agree that the assumptions about measurement noise are tricky – under many real-world conditions the relationship between process and measurement noise is difficult to untangle and can have a strong influence on the assumed stability of a system. To understand how measurement noise influences λ estimates, we take a fixed process noise level ($\sigma = 0.01$) and add variable amounts of white noise to the synthetic time series (Figure 5).

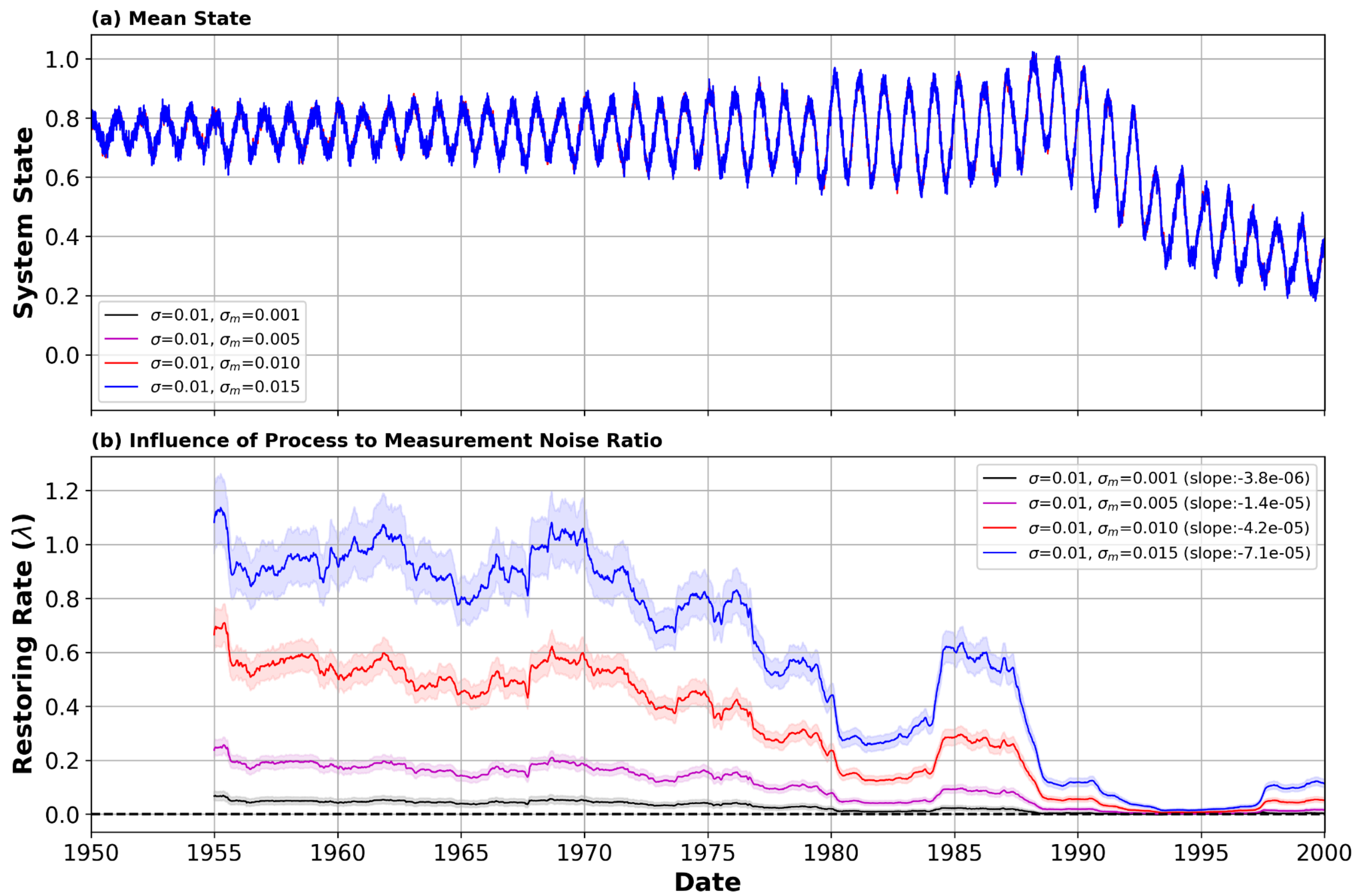

Figure 5: The influence of changes to measurement noise on the inferred λ.

We find that the functional form of the λ changes is maintained, but, as expected, the absolute value of λ shifts substantially. Trends are still negative across realizations, with slopes varying with λ magnitude. Under constant measurement noise, we do not find biases in λ trend directions or in our interpretation of how changes in λ relate to changes in system stability.

For small measurement noise, this impact is expected to be negligible, as we note in the paper. As measurement noise grows, however, λ will be systematically biased high – especially if measurement noise exceeds process noise (Figure 5). We note that the direction of λ bias was inverted in the original draft of the MS, and has been corrected in our Revision (bottom of p4).

As a related test, we compare realizations of our same synthetic series with different process noise levels (Figure 6). We find that increasing process noise levels systematically increases λ, though it does not unduly bias the λ trajectories.

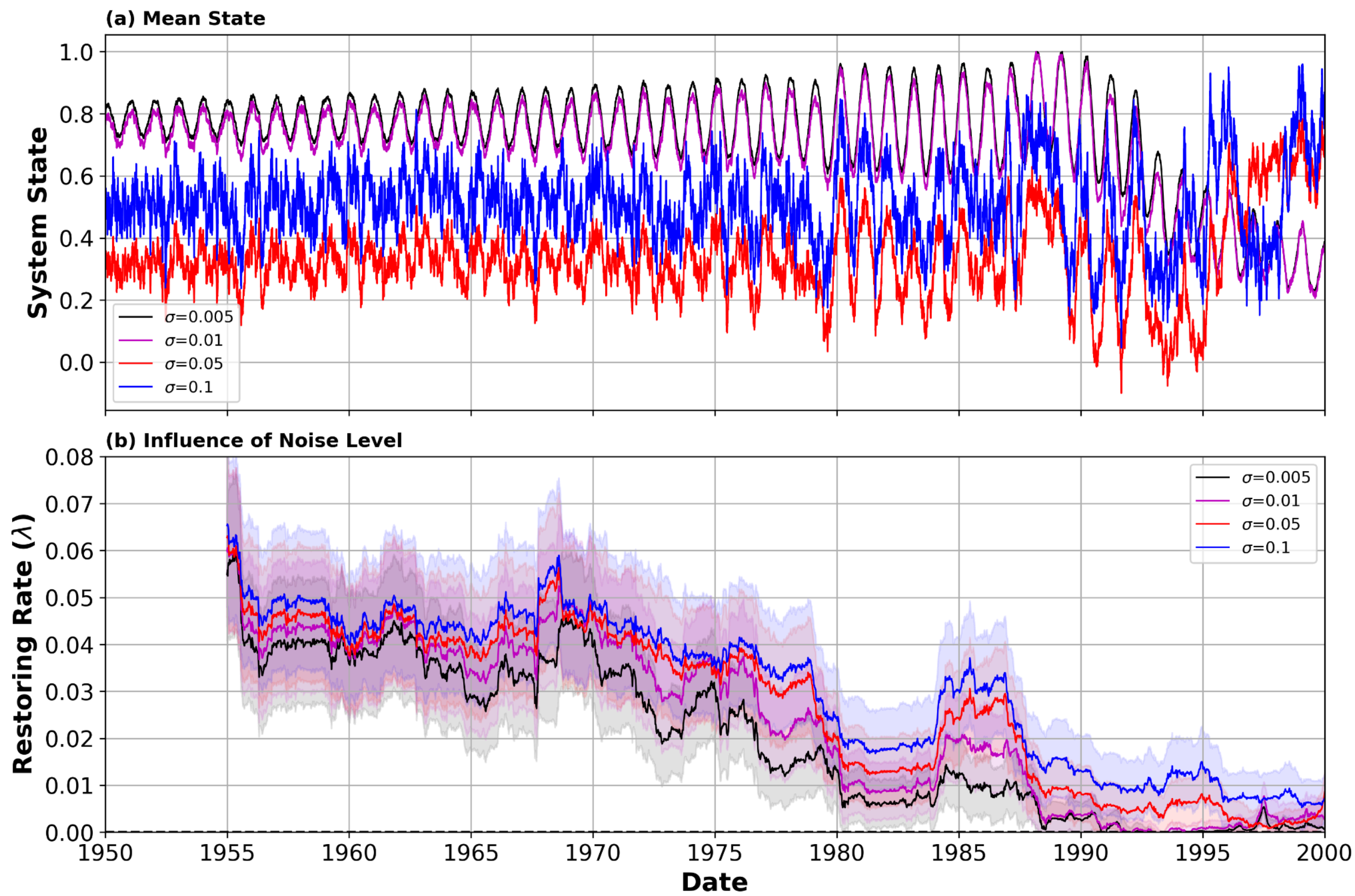

Figure 6: The influence of changes to the process noise level on the inferred λ.

We have added a discussion of this point to the manuscript, alongside an additional Supplemental Figure.

3. Attractor Model Misspecification
The framework requires the user to specify a functional representation of the attractor dynamics through the design matrix (e.g., Fourier components or polynomial trends). A natural concern is whether misspecification of the attractor model introduces biases into the estimated restoring rate. For example, if the true attractor contains higher-frequency components or nonlinear structures not represented in the chosen basis, can these unmodeled components leak into the resilience estimate?

The authors should discuss this issue more explicitly and, if possible, provide a synthetic example illustrating the impact of attractor misspecification on the inferred restoring rate.

This is a great comment – indeed, our method will be sensitive to misspecified attractors, and this should be further discussed in the MS. It is possible to construct such an example by adding, e.g., additional harmonic terms to the synthetic system without adding additional harmonic terms to the regression (Figure 7), or simply by leaving the harmonic terms out of the regression design. We find that misspecification in this case leads to λ being biased low as there is substantial variability left in the data that is not captured through the harmonic terms – this

would also be the case for, e.g., computing AC1 on a time series with a linear trend. This is less of an issue when the attractor is misspecified with too many terms (e.g., three harmonics for a two-harmonic system), as the additional (unneeded) terms absorb relatively small amounts of variability.

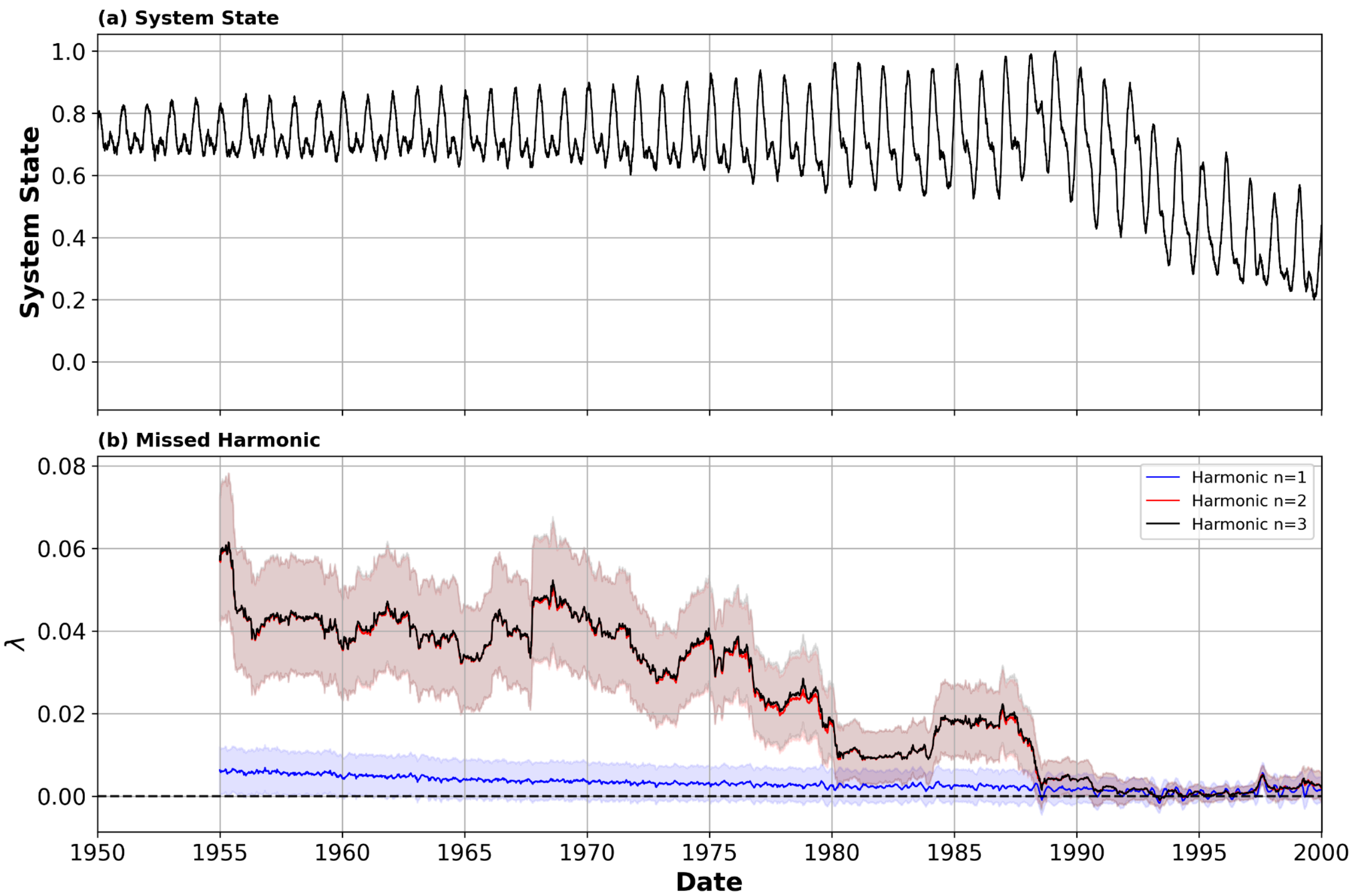

Figure 7: Synthetic model with an additional harmonic term (annual and semi-annual harmonics), and the resulting λ estimates when that additional harmonic is specified in the design matrix or left out.

We can also test this on a more common issue – a linear trend in the data that is not properly removed or accounted for in the λ estimate (Figure 8). In this case the bias is relatively smaller, as the third-order harmonic can to some degree absorb a missed (small) linear trend, leading to a smaller negative bias in λ.

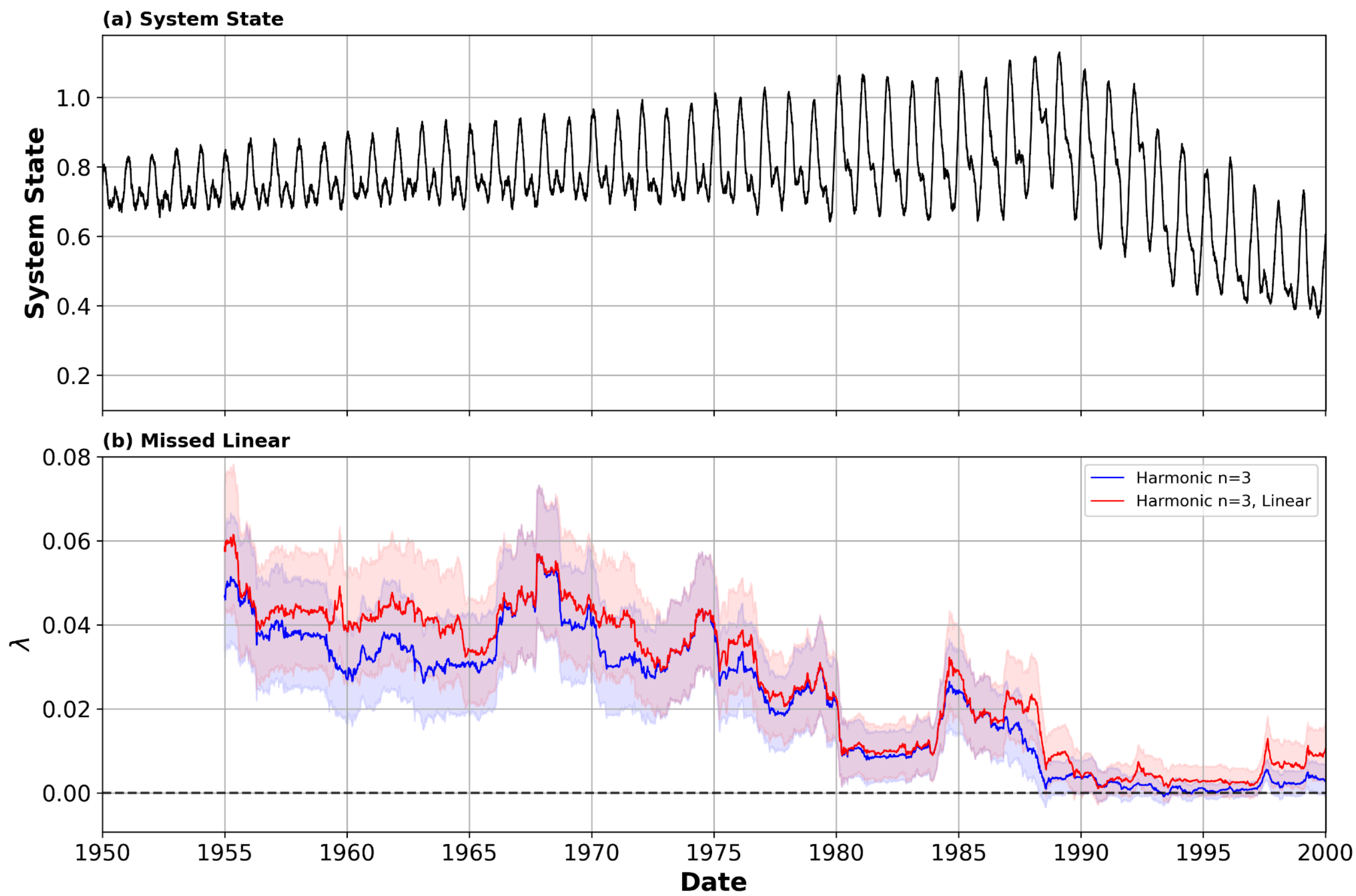

Figure 8: Synthetic model with an additional linear trend, and the resulting λ estimates when that linear trend is modeled or left out.

There will always be some danger of model misspecification leaking into the λ estimate when we do not explicitly know the forcing. There is relatively less danger of biasing λ when too many terms are included in the design matrix, as the robust solver can assign these terms very low weights (e.g., a linear term in the design matrix is not needed to explain the movement of a flat line); there remains, however, danger of overfitting if too many terms are included, especially higher order (e.g., quadratic) terms. If the physical process driving the system is to some degree known (e.g., vegetation follows an annual seasonal cycle with n=1,2,3 harmonics), our λ estimates have a strong physical basis. If the shape of the attractor is unknown, there is relatively more danger of misspecification and biased λ estimates, as it would not be clear in that case which parts of the signal should be partitioned into the attractor and which into λ.

We have added a discussion of this point to the Discussion section of the MS.

4. Cross-Validation via Kramers–Moyal Coefficients

The proposed regression framework can be interpreted as a parametric estimation of the first Kramers–Moyal coefficient D(1), corresponding to the deterministic drift term of the underlying stochastic process. To further validate the methodology, I recommend that the authors compare their estimates with standard non-parametric Kramers–Moyal reconstruction techniques. In particular:

- Estimate the first Kramers–Moyal coefficient using the kernel-based approaches on synthetic datasets.
- Compare the local gradient of the reconstructed drift function with the restoring rates obtained from the regression framework.
- Demonstrate consistency between the parametric and non-parametric estimates.

Such a comparison would establish a direct connection between the proposed method and the classical stochastic-process framework underlying Langevin dynamics.

Thank you for bringing this up – it is indeed possible to compare the two frameworks, though there are some limitations to Kramers-Moyal that make a direct comparison difficult. The main difficulty is that Kramers-Moyal coefficients cannot be estimated directly on seasonal data – the data must be deseasoned/detrended before a reliable statistic can be derived. This creates some of the same issues that motivate our approach: confusion around the impact of deseasoning techniques. If we apply three common deseasoning/detrending schemes (cf. Figure 1 of the MS), we can calculate the KM D(1) coefficient for comparison (Figure 9).

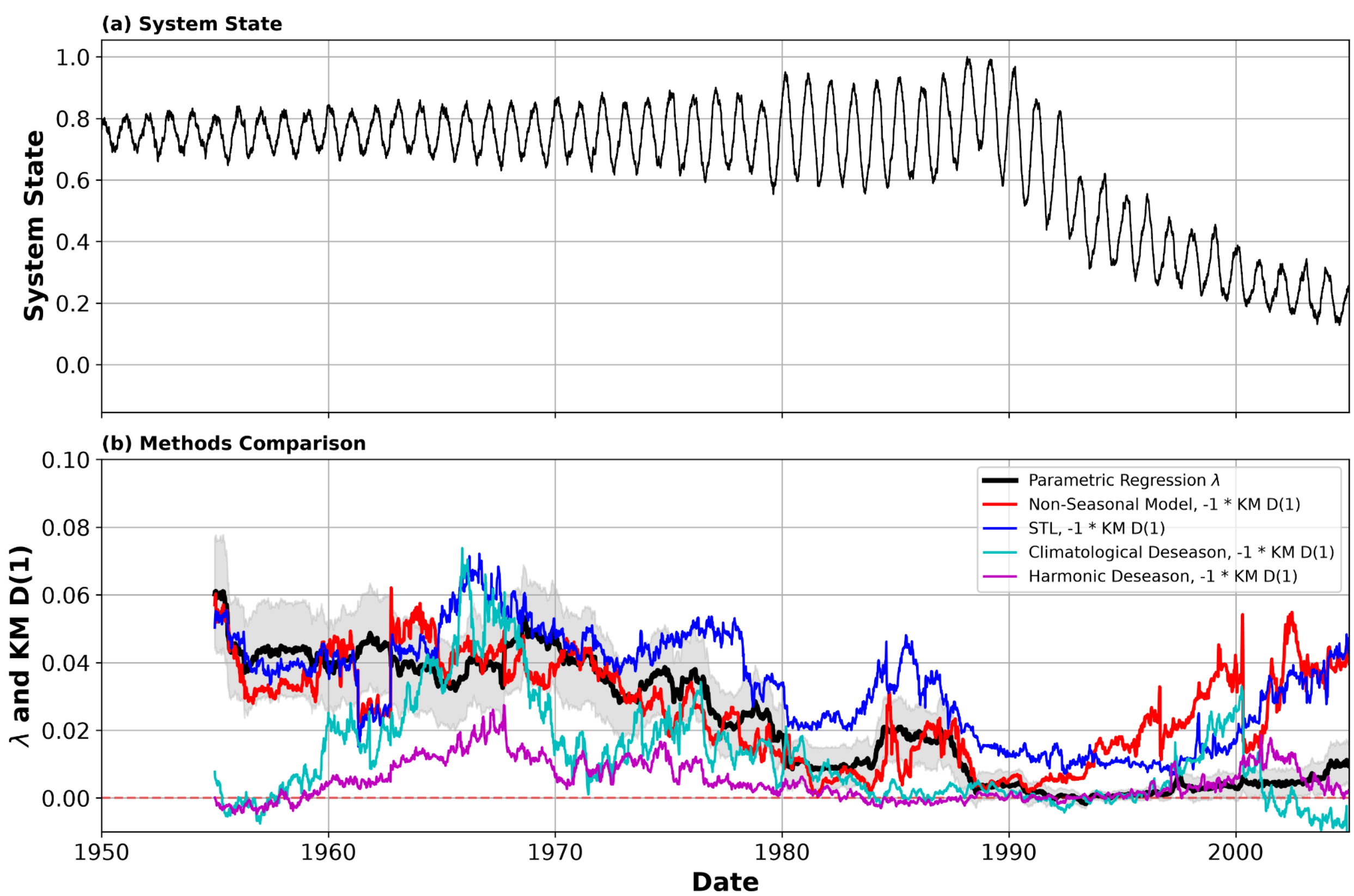

Figure 9: Kramers-Moyal D(1) coefficients computed using three different deseasoning/detrending techniques and against a non-seasonal run of the same synthetic model. KM coefficients scaled with -1 to match the direction of λ changes.

We find that for the non-seasonal model and STL deseasoning, the KM D(1) coefficient matches our λ relatively well, though it does not approach as close to zero for STL. The other two

deseasoning techniques show decreases towards the transition point, but also have relatively low KM D(1) values at the start of the time series. This is qualitatively what we see in Figure 1 of the MS, where the λ estimated with climatological or harmonically deseasoned data is biased strongly low at the start of the time series.

We note that KM D(1) statistics do not solve some of the core problems our method addresses: direct application on non-stationary and unevenly sampled data without extensive preprocessing. We have added a note to this effect in the Theoretical framing section.

5. Non-Parametric Benchmarking Using Kernel Regression
The methodology relies on a predefined representation of the time-varying attractor. Although the authors demonstrate robustness for several examples, it remains unclear how sensitive the results are to the specific basis functions chosen. As a robustness check, I suggest employing a non-parametric benchmark such as Nadaraya–Watson kernel regression or local linear regression to estimate the evolving attractor.

The resulting resilience estimates could then be compared with those obtained from the proposed harmonic or polynomial design matrices. Agreement between the two approaches would substantially strengthen confidence that the inferred resilience trends are not artifacts of the chosen functional form.

Thank you for this excellent point – this is indeed something we have also thought about, and were preparing for a future publication. A full treatment of non-parametric methods is outside the scope of this paper, but we can provide some first-order comparisons and a short discussion around the advantages / disadvantages of the parametric and non-parametric approaches.

Non-parametric (e.g., kernel, Nadaraya-Watson) approaches help to avoid misspecifying the attractor form, but they introduce other issues that are not present in the parametric (e.g., design matrix) approach. The most critical drawback is separation of the attractor and the stochastic fluctuations – if the kernel bandwidth is set very narrow, it is likely that small fluctuations (i.e., the autocorrelated noise we want to measure) are treated as part of the attractor itself, and key signal is lost. This is also something that can happen during deseasoning (e.g., if too little or too much season is removed from the signal), and avoiding such signal misclassification or overfitting is a motivation for our current parametric approach. If we compare two non-parametric methods to our parametric method, we can see that λ is strongly biased low (~10% of the parametric estimate) (Figure 10).

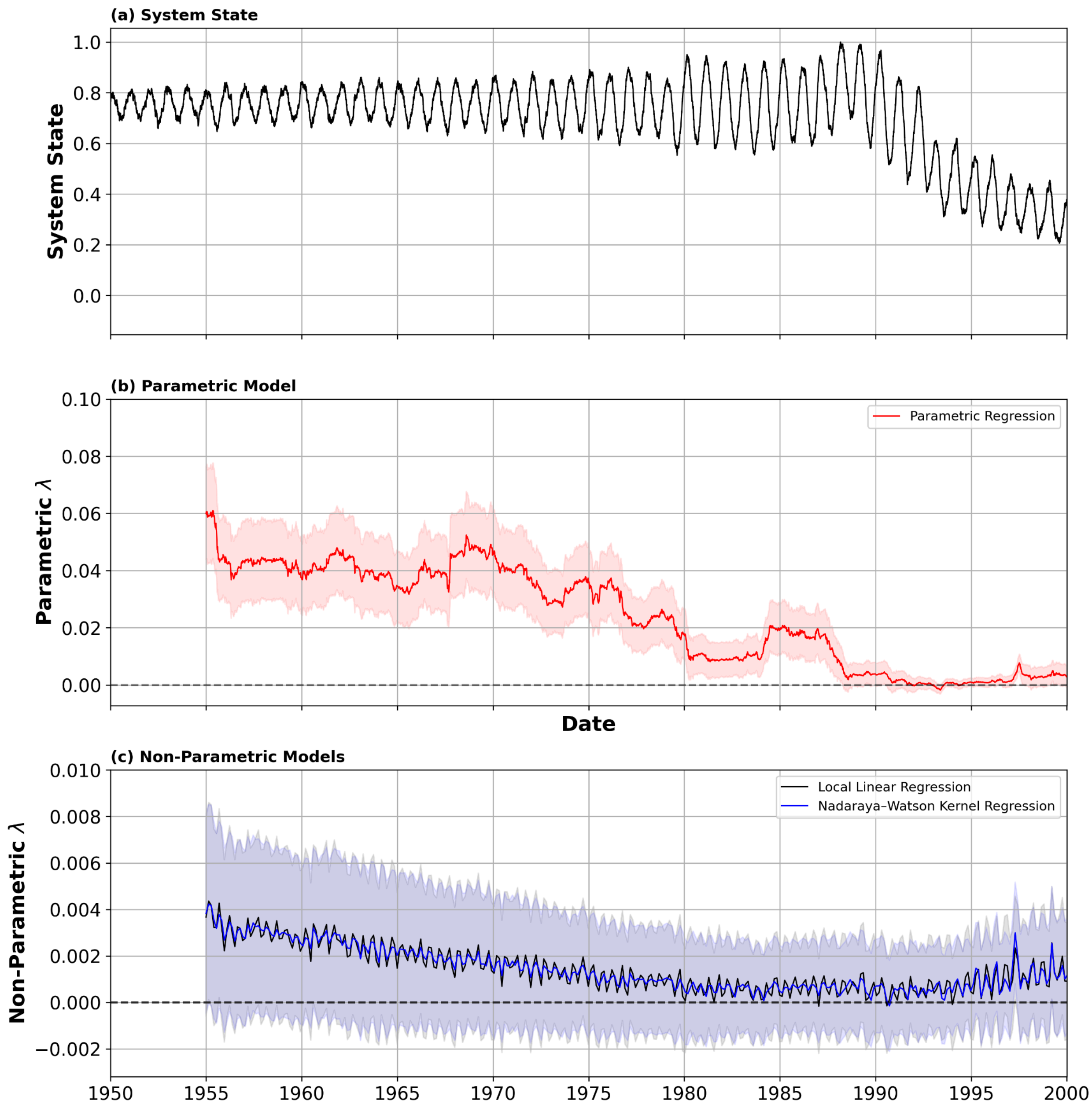

Figure 10: Comparison of parametric and non-parametric methods for estimating λ. Non-parametric methods are generally biased low.

The same issue of 'overfitting' can also be thought of as signal leakage – in our parametric approach, we specify that the signal is made up of internal variability, seasonality, trend, and some residual noise. This enforces a desired signal partitioning (via our regression design matrix) around that known reality. Non-parametric methods do not know this partitioning a priori and can lead to, e.g., a linear trend being absorbed into 'seasonality', biasing the estimate of λ.

While both parametric and non-parametric approaches require some hyperparameters to be set (e.g., the order of annual harmonics), it is much easier to physically motivate these parameters

for a parametric approach – the harmonic order for annual seasonality is fairly straightforward, while the bandwidth choice for a non-parametric approach is much more difficult to select. Misspecified bandwidth – both too high or too low – can influence how well the model fits the data, and hence the λ estimate (Figure 11).

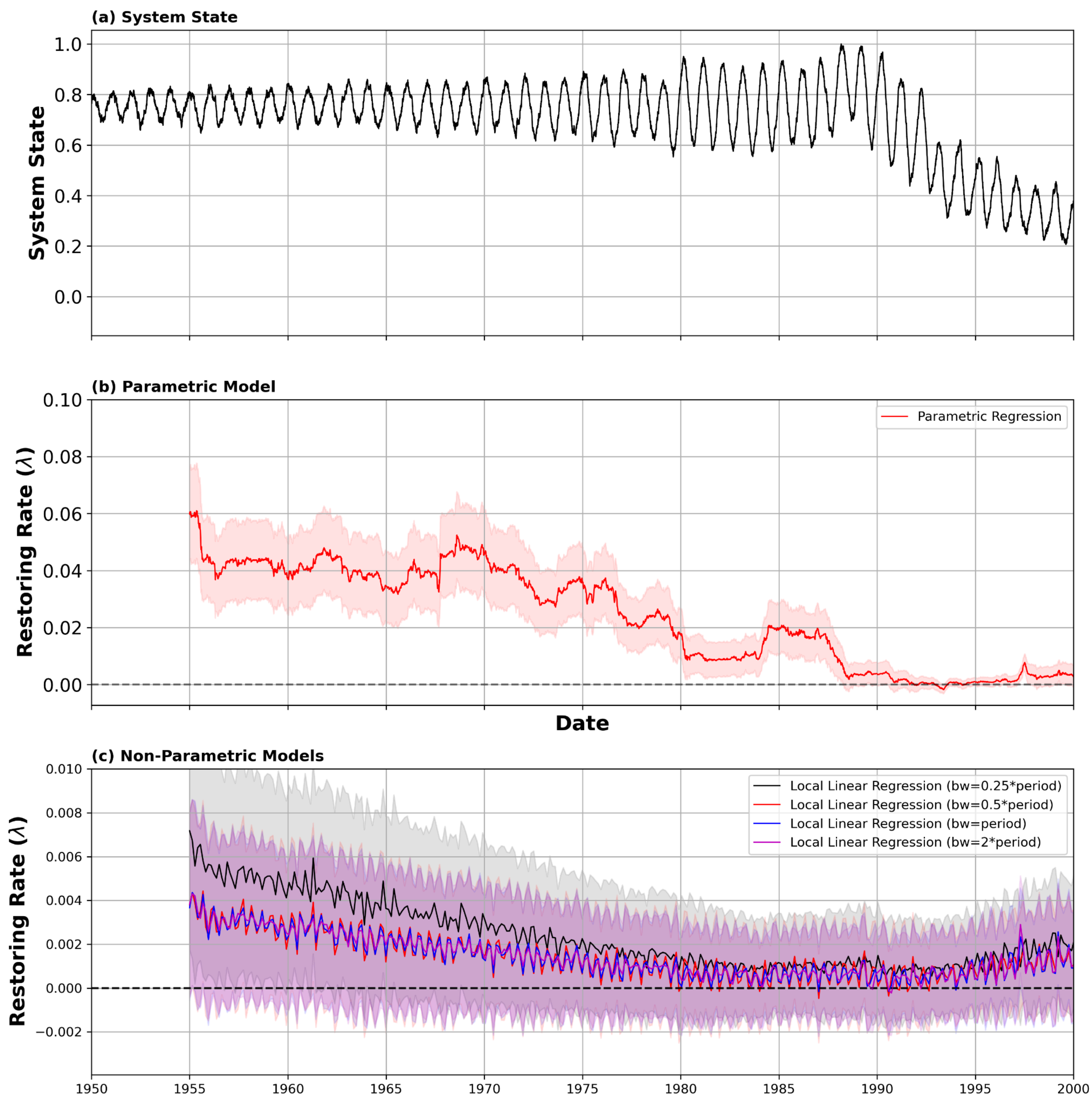

Figure 11: Influence of bandwidth selection on the magnitude of λ estimated with a local linear regression. In all cases, there remains substantial high-frequency jitter in the λ estimates.

With regards to data gaps – a parametric model is to some degree constrained and can handle missing data (e.g., see Figure 1). This is not the case for non-parametric models, where data gaps can bias the estimated functional form and hence bias λ. That is not to say that parametric models are immune to the influence of data gaps, but rather that they have a more solid

‘fallback’ in the known functional form than non-parametric methods, which must find a local attractor for each new set of data.

The last two drawbacks of non-parametric approaches are (1) speed and (2) interpretability. It is clear that fitting a regression with a set design matrix is a fast operation – running this on daily data for decades takes a few seconds. This is not the case for non-parametric methods, which scale very poorly with data density or the number of moving windows (the same time series can take hours to process, though vectorizing the approach brought this down to ~20 minutes in our tests). This limits their use in, e.g., large ensembles of time series like those shown for global vegetation data in Figure 2.

The drawback regarding interpretability is more qualitative, but remains important. When the estimation of λ is specified directly with regards to some physical constraints (e.g., known annual seasonality and slow linear trend), the resulting λ is directly physically interpretable. Non-parametric methods, on the other hand, return changes in λ relative to some local moving average or local kernel, which is harder to attribute to a physical driver or process.

In this paper, we focus on our constrained parametric approach for real-world data. We have added, however, non-parametric estimators to our framework. In short, we use Nadaraya-Watson and local linear regression to construct non-parametric local state and dx/dt estimates. Subtracting those components allows us to create state and dx/dt anomalies, which can then be fed into our same regression framework as a zero-intercept linear regression problem. We can resolve this regression using the same methods as with our parametric approach (e.g., robust regression).

We note, however, that the non-parametric approach is essentially a sequential, two-step estimation process (as opposed to our simultaneous, one-step parametric regression) which treats the derived anomalies of x and dx/dt as error-free observations, and does not propagate estimation variances forward into the confidence intervals of λ. Properly accounting for that uncertainty is analytically challenging – especially when the kernel bandwidth is selected automatically – and estimating confidence intervals via bootstraps is computationally prohibitive over many moving windows. More complex one-step procedures (e.g., using varying-coefficient models) could in principle mitigate this bias, but would be very difficult to correctly parameterize for real-world data. Hence, we present a simplified non-parametric framework here for comparison with our parametric approach, but note that the confidence intervals of λ we present are likely too narrow (Figures 10,11).

We include a longer discussion around non-parametric approaches and, in particular, how they may help when the functional form of the attractor is not known, to our Discussion section, and have added a complete description of the non-parametric anomaly approach to the Methods and our codebase on Zenodo.

6. Comparison with Alternative Reconstruction Frameworks
The global vegetation application represents a high-dimensional spatio-temporal system in which spatial interactions and network effects may play a role. For this reason, it would be informative to compare the reported resilience trends with those obtained using an alternative dynamical reconstruction framework such as HINTS (HiNTS: Higher-Order Interactions in N-Dimensional Time Series) or related moment-based reconstruction techniques.

While a full analysis may be beyond the scope of the current manuscript, even a limited comparison on a subset of the vegetation dataset would provide an independent validation of the inferred resilience loss and demonstrate consistency across reconstruction methodologies.

Thank you for pointing out this method, and the general framework for moment-based reconstructions. This is a really interesting alternative way to get at stability based on building probability distributions from higher-order moments rather than Jacobian-based geometry. As it is primarily useful in an nD case (i.e., the higher-order moments of the 1D time series shown here for vegetation collapse to, e.g., skewness), a direct comparison to our results in Figure 2 is difficult. For the vegetation data shown here, we work on individual time series independently, and do not capture any spatial interactions.

A closer comparison would be to our results in a previous paper which used Dynamic Mode Decomposition to estimate stability using Floquet Multipliers over spatio-temporal data fields (https://doi.org/10.1038/s41467-026-68944-w), which seems like a much more direct cognate of the HiNTS framework. Any comparison would have to be carried out over defined spatial areas due to both physical interpretability considerations and computational efficiency – computing massive interaction matrices on dense spatio-temporal data is not straightforward. It seems like the HiNTS framework was designed for much different data sizes and dimensionality than what we typically work with in global vegetation: a spatial field of millions of pixels. Even for our synthetic vegetation model (50 x 50 pixels), a full multivariate moment expansion rapidly becomes underdetermined when considering higher-order interactions, given the limited number of independent temporal samples available within moving windows (e.g., 1095 for 3-year windows of daily data). For this reason, any application of HiNTS to global vegetation data must necessarily rely on a reduced representation of the system.

Looking at the HiNTS github, it seems like we may be able to motivate a more limited comparison based on the synthetic vegetation model we use here. In our λ estimate via regression, we treat all samples as independent – we perform our regression by collapsing the spatial dimension and using all points in a pooled regression. To avoid the abovementioned data problems in implementing HiNTS, we tested a simple data subsampling routine to create a reduced representation of our synthetic vegetation data by taking the first PCA alongside a sparse set of random samples (n=5,10,25) in order to capture at least a bit of the spatial interaction in the model. We applied the Kramers-Moyal reconstruction procedure from HiNTS (https://github.com/aminakhshi/hints/blob/main/hints/hints.py) on that data, and compared it to our regression-based λ.

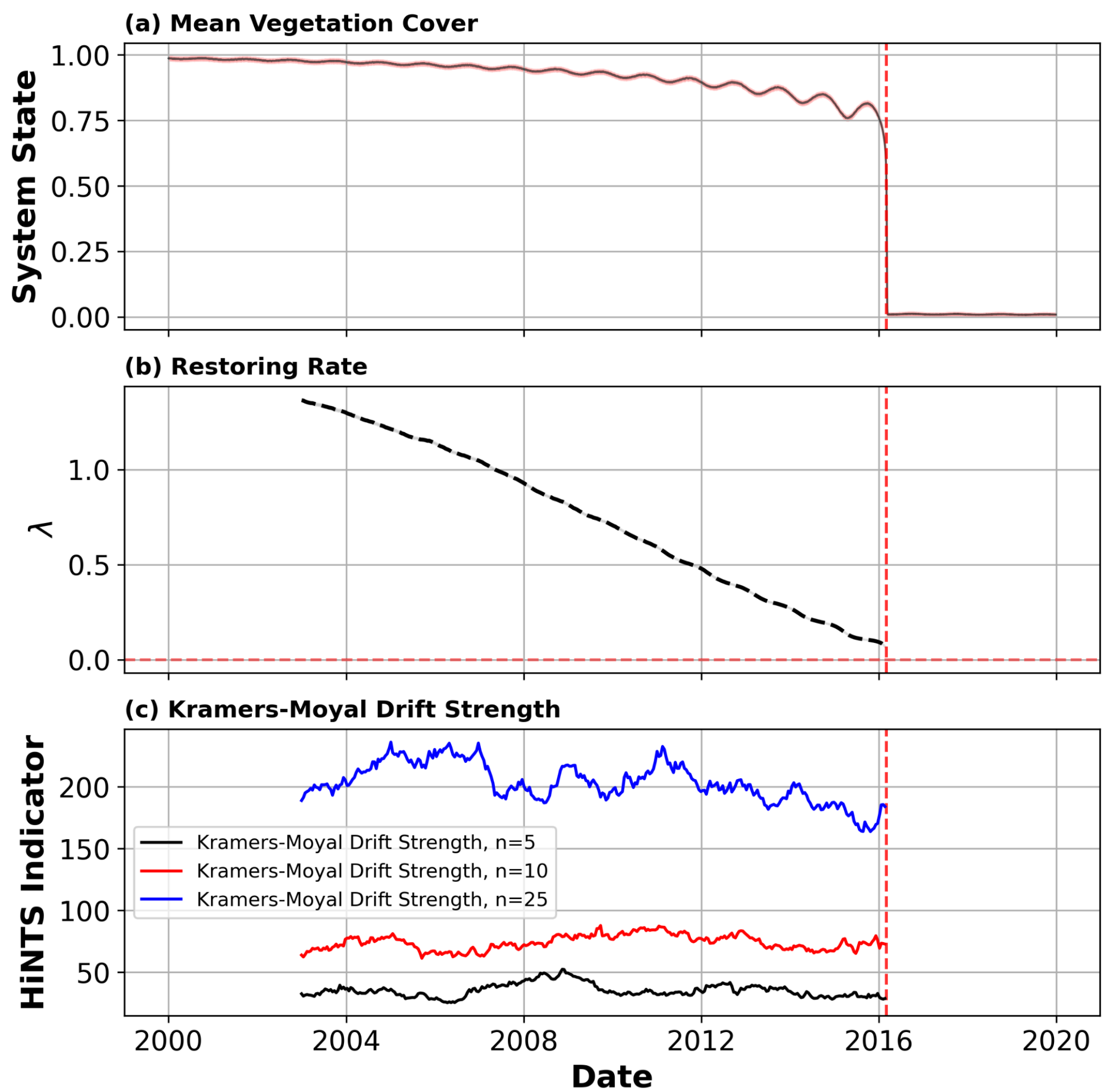

Figure 12: Comparison between λ and Kramers-Moyal drift indicators on a reduced basis of the spatial vegetation model (Supplemental Figure S2).

We plot the norm of the linear drift coefficients in Figure 12, finding unclear early warning signals before the vegetation state change. We note that we tried using higher n (n=50, n=100) and the Kramers-Moyal coefficients blew up to the order of 1e6 – we attribute this to an insufficient number of independent samples in the moving temporal windows to resolve the interaction matrix. The indicator is numerically stable for small n, but not for anything above ~n=30, which we take as further confirmation that moment-based reconstruction will be difficult for short and non-stationary time series that are typical of satellite vegetation data.

We hesitate to directly equate the two metrics, as they capture different parts of the system dynamics. Our λ estimate is derived from the local linear stability of the full spatio-temporal field (via pooled regression), and the HiNTS-based indicator rather reflects the structure of a fitted stochastic drift field, which depends on both the system projection (i.e., into lower dimensions) and the interaction between (randomly chosen, in this case) vegetation pixels. This encodes

rather the nonlinear interaction structure than the direct linear stability – both approaches can capture changes before a state transition, but they do not measure the same thing.

As the reviewer notes, a full comparison is beyond the scope of this manuscript. We would be interested to further explore how HiNTS and related reconstruction methods could be used in the context of global vegetation data, however – that is a great idea for a future research direction.

7. Robustness to Noise Structure
The derivation assumes Gaussian white noise forcing. However, many Earth system records exhibit temporal correlations, heavy-tailed fluctuations, or state-dependent noise. I encourage the authors to discuss the extent to which their restoring-rate estimates are affected by:

- temporally correlated (red) noise,
- non-Gaussian forcing,
- multiplicative or state-dependent noise.

Examining the behavior of the second Kramers–Moyal coefficient D(2) in the real-world examples may help determine whether diffusion effects influence the reported resilience estimates. Such analyses would significantly strengthen the generality and robustness of the proposed framework.

Thank you for this comment. Indeed, the noise structure can play a large role in how λ is estimated, and red noise is a particular problem for many CSD methods. Our method is to some degree already conditioned against strong noise in that we use a robust regression including weights that can account for some kinds of noise – such as multiplicative or non-gaussian noise – quite well. These kinds of noise would be much harder to deal with in standard ordinary least squares (i.e., using the 'naive' regression included in our python code). To test the impact of different kinds of noise, we adapted our synthetic model to use five different noise forcings: (1) white, (2) red, (3) heavy-tailed (Student-t), (4) skewed (Skew-normal), and (5) multiplicative (state-dependent).

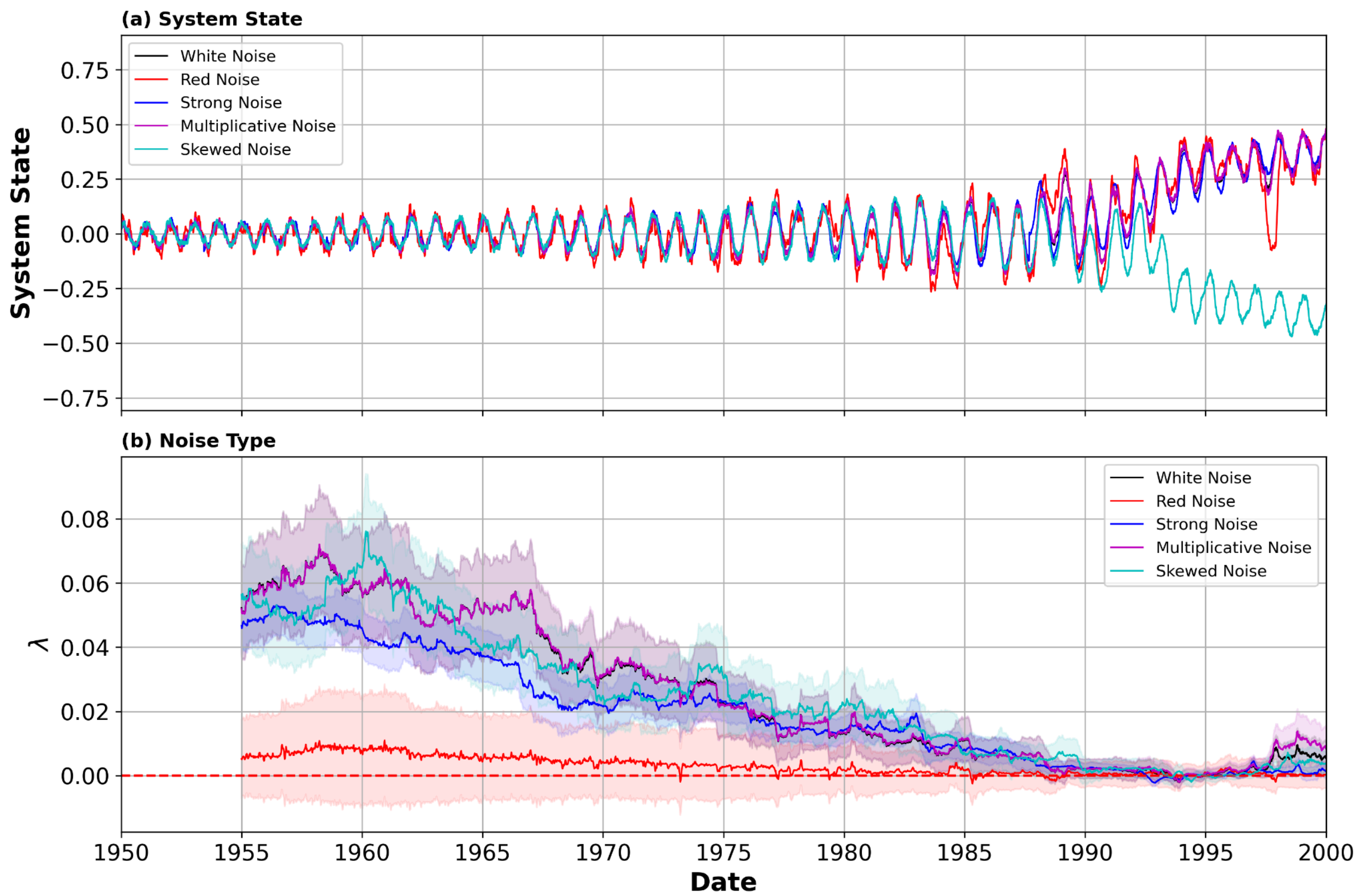

Figure 13: Influence of process noise structure on the inferred λ estimates.

For all noise models – including state-dependent noise – changes in λ are still captured, albeit with slightly different magnitudes. Strong and non-Gaussian noise is not an issue in our framework – the robust regression approach effectively minimizes issues around noise amplitude. Red noise remains an issue, however, leading to drastically smaller λ estimates. We still capture the *drift* in λ towards zero, though with a smaller amplitude and error bounds that encompass zero from the start.

Fortunately, this is also a problem that can be directly addressed within our framework – we include a GLSAR regression that handles correlated noise in our code which can be substituted seamlessly for the default robust regression. Replacing the regression model yields improved results for red noise (Figure 14), which are quite similar to the results for white noise.

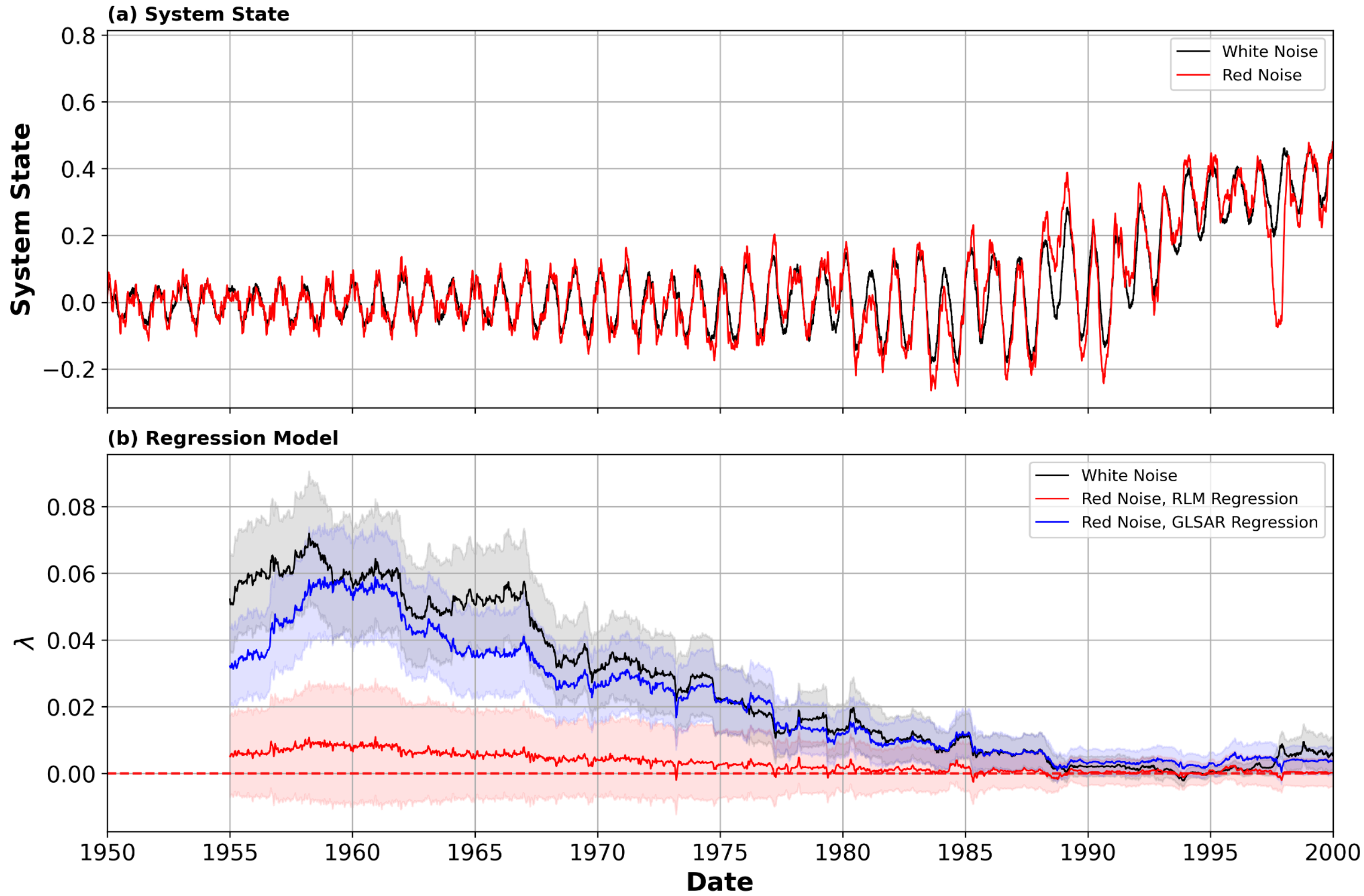

Figure 14: Influence of changing the regression specification for red noise. For known red noise, a GLSAR regression performs better than a weighted robust linear regression.

The second Kramers-Moyal coefficient D(2) is not straightforward to compute on real-world data, especially data with seasonality and irregular time steps. First, measurement noise will accumulate to varying degrees over irregular step sizes; as D(2) depends directly on the variance, it can dramatically increase for small step sizes. Second, as with the D(1) calculation (Figure 9 of this Reply), the data must be deseasoned and detrended beforehand – this is precisely one of the core motivations for our parametric regression approach, in that it does not require such preprocessing.

For the global vegetation data, deseasoning/detrending each individual time series and examining the D(2) coefficients is outside of the scope of this study, but would be interesting to, e.g., examine differences between different land covers. The glacier data presented here was chosen in part because it is so difficult to deseason, and hence stability estimates relying on, e.g., STL deseasoning are problematic. The paleoclimate data is thus the one real-world example we include which could in theory be analyzed with the D(2) coefficient. However, the large and irregular sampling interval alongside substantial proxy noise makes distinguishing between statistical artifacts and real state dependence in the noise structure difficult. If we compute the D(1) and D(2) coefficients for our ice-core data, we find a curved relationship between D(2) and the system state (Figure 15).

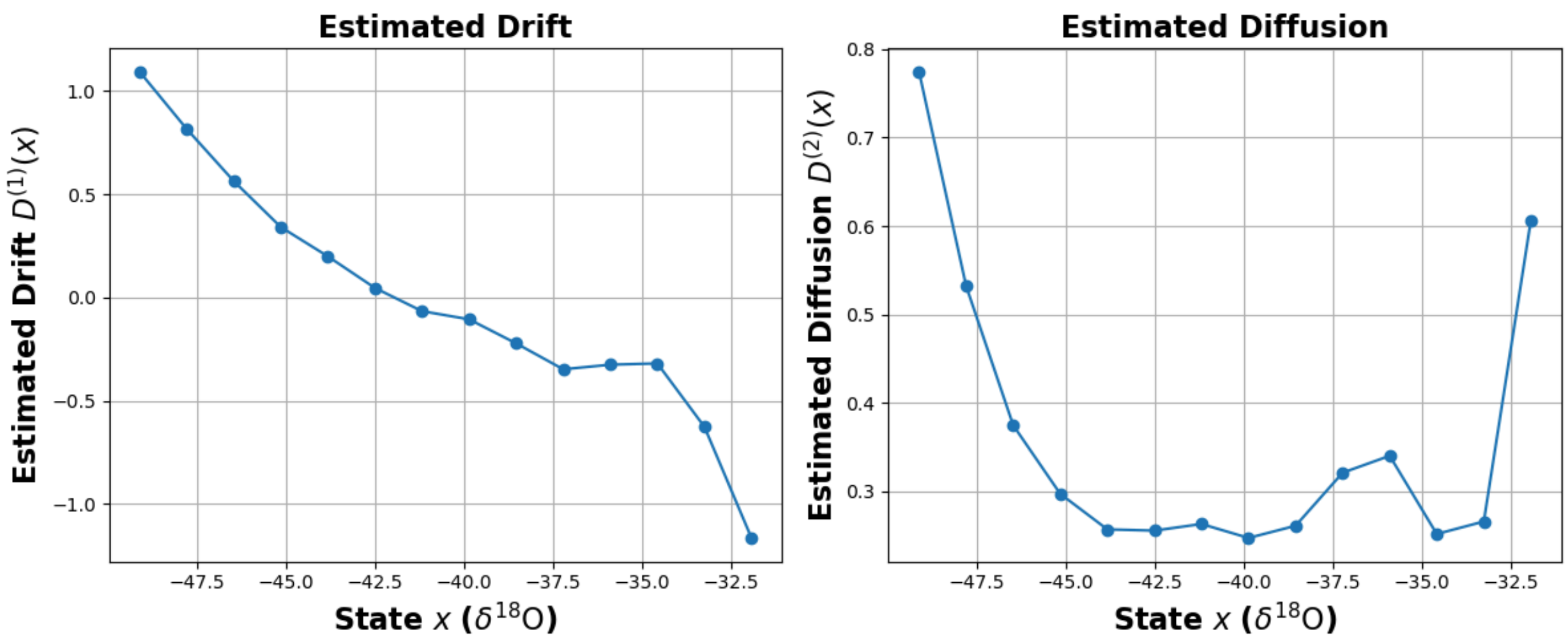

Figure 15: KM Drift and Diffusion coefficients for the paleoclimate proxy data used in our study.

The linear drift-state relationship is expected, and has been shown by other authors (e.g., Riechers et al., 2023, https://doi.org/10.5194/esd-14-593-2023). The remaining question is whether the U-shape found in the diffusion-state relationship is a data artifact or due to the noise structure itself. Though we applied the leading order corrections in the finite-time estimation of the Kramers-Moyal coefficients, higher-order error terms are likely to be one reason for this particular shape (https://doi.org/10.3390/e23050517). Beyond this, we manipulate the data in distinct ways to isolate further sources:

We can perform a simple first-order analysis by (1) linearly detrending the paleoclimate data, (2) resampling the data to an even time step to see if the sampling regime produces the U-shaped curve, and (3) generating a flat-noise surrogate and sampling it at the exact GICC05 ages in our paleoclimate data (Figure 16).

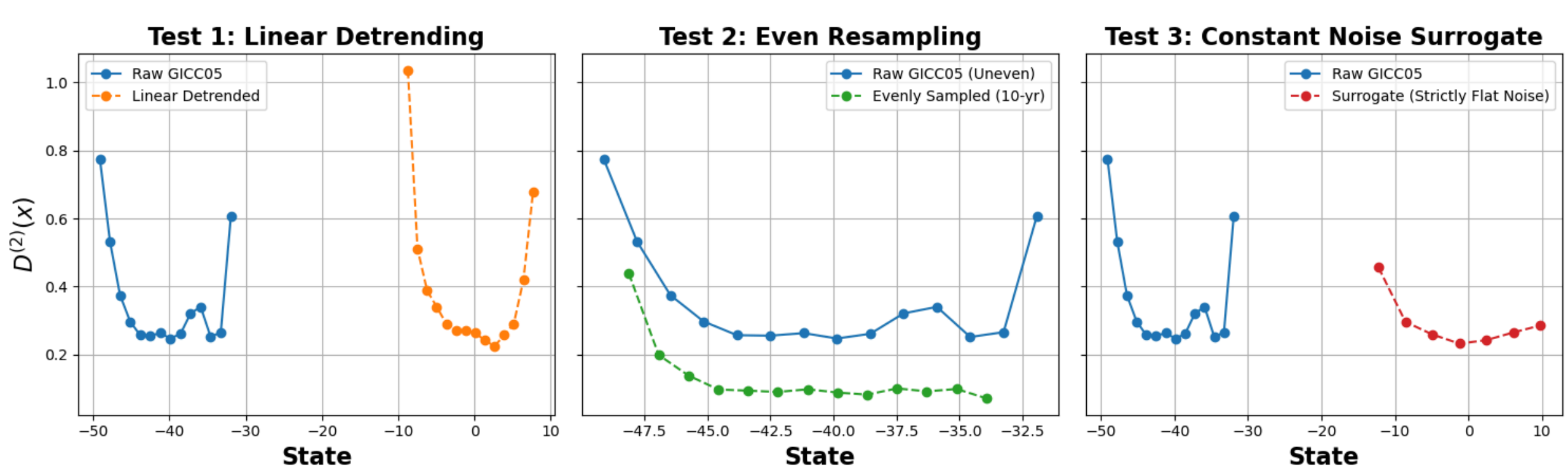

Figure 16: KM Diffusion-state relationships for different surrogates (detrending, resampling, constant noise).

We find that linear detrending only serves to relocate the U-shaped curve centered at zero, and does not flatten the curve. Resampling the data to an even 10-year time step to some degree flattens the curve, and removes the upwards trend for high state values. Finally, reproducing the

sampling structure of our data from a process with constant noise also produces a U-shaped curve, indicating that the diffusion-state relationship is to some degree controlled by the uneven sampling in the paleoclimate data. We thus conclude that the underlying climate noise is state-independent; this confirms the results of Riechers et al. (2023) as well. We further note that the vast majority of data points live in the state space regime that exhibits constant noise strength, and, as such, we do not anticipate large impacts on our results.

We cannot directly comment on the state-dependence of the noise for the glacier or vegetation systems, but consider this an interesting avenue for further research. We note, however, that our framework can flexibly handle different kinds of noise, including state-dependent noise, without biasing our λ estimates (Figures 13, 14 of this Reply). We thus provide a framework for analyzing systems with different noise regimes, and would encourage thorough investigations of the noise regime before applying our method, when such investigations are feasible. We include a note about noise regimes in our updated Methods section.

Minor Comment

1. A brief discussion of computational efficiency would be useful. Although the proposed method is clearly more sophisticated than standard AC1 estimation, readers working with large spatio-temporal datasets would benefit from an indication of computational scaling and runtime.

Thank you for this suggestion – we have included a note about this in the Methods section. In general, linear regression is very cheap, and has the added benefit of not requiring pre-processing (e.g., deseasoning). For the synthetic data shown in Figure 1, the regression approach takes ~11 seconds to run, while AC1 takes ~9 seconds for deseasoning/detrending with STL and ~1 second for the AC1 calculation itself. Our approach is thus slower by ~1 second, which, depending on how much data needs to be processed, is a reasonable extra expense.

Recommendation

Recommendation: Accept after Minor Revisions.

The requested revisions primarily concern additional validation, robustness analyses, and clarification of methodological assumptions. These suggestions are intended to further strengthen an already strong contribution rather than to address fundamental shortcomings.

Thank you for your extensive comments – they have given us several important new points to raise in the MS and filled some critical gaps in our argumentation.

Reply to Reviewers - R2 - Smith et al, Nature Communications

Reviewer #1:

The authors have made a concerted effort to address the concerns raised in the first version. Unfortunately, I now have more concerns, given I understand more of what is in the manuscript.

Thank you for taking time to re-review the work. We have answered each of your comments in-line below.

(a) The only details of the theoretical "pitchfork" model used to explore the method seem to be in the supplementary information, where it is said: "For the pitchfork-bifurcation time series model, we use the same model setup as was used in Smith et al. (2026)." However, there is no such reference – I assume they mean equation (3) of [13] Smith et al (2025), which is now published in Nature Comms (2026). It seems they did not update this in the revision. This is quite inadequate given that this "basic model" is explored in detail in many of the figures and is a key motivating example. I think at least the equation should be given.

The equation for the model is found in Equation 19 of the Manuscript (in the Methods, under 'Synthetic Time Series Data'); we also include text description of the model there. We include the exact code to generate the model in the code repository (https://doi.org/10.5281/zenodo.19731233), and list the parameters used in the Supplement as well (page 2). You are correct, however, that the Zenodo link in the previous Supplement still pointed to the Zenodo page for our previous publication – this link has been updated in the new submission, as we have compiled all code needed to reproduce the main manuscript and Supplemental Figures for this paper in one Zenodo archive.

(b) In spite of (a), I have a growing concern about the amount of overlap with Smith et al (2026): it is not clear to me that the conclusions about the glacial surging or ecosystem tipping are much of an advance on what was found in Smith et al (2026) with other methodology. The methodology is, however, still interesting and deserves to be published.

Thank you for this comment. Indeed, many of the conclusions are similar – the changes in the dynamics captured by multiple means of estimating λ should (ideally) be the same across methods. In the previous work, complex state-space embedding was needed, making the method more difficult to apply generically across many time series. This work removes that condition, adds robust uncertainty propagation, removes the reliance on evenly sampled and gap-free time series, and provides a 'drop-in' replacement for commonly used AC1-based resilience estimators. We feel that this is a substantial addition to the literature, in that it provides a well-motivated method of avoiding many common data issues (e.g., preprocessing, uncertainty propagation) that crop up across disciplines. We do not claim strong novelty in the applications; they were chosen to illustrate how the method can be applied across diverse real-world systems, and not to break new ground in the types of systems studied using dynamical

systems theory. Our work rather provides a strong basis for future analyses of new systems and applications.

(c) I have concerns about the relevance of this theoretical “pitchfork”. As the case considered is supercritical, in some definitions this would not be seen as a critical transition – the attractors after bifurcation are close to those before, and so I can’t see an argument that this is modelling an “abrupt change”. This is not a very challenging example given that there is no trend that needs to be removed before the bifurcation – we are fortunate to be near an unchanging equilibrium in the absence of noise and seasonal forcing. I would expect to see evidence of more than one attractor after the bifurcation, unless the noise and forcing are carefully chosen to always end up on one branch, and possible hopping between these attractors. Is there any example in the climate literature where such a pitchfork is found?

We did not design our synthetic model to represent any real-world system, but rather to provide a minimal toy model for non-linear bifurcation dynamics. The essential characteristic of a dynamical system approaching a bifurcation is the loss of its linear stability. The pitchfork bifurcation offers a test case in which no competing stable state and no drift of the stable equilibrium exists, placing the sole focus on the detection of critical slowing down. The lack of an abrupt shift after the bifurcation does not impact the aptness of the model as a benchmark for our method’s ability to discern a loss of linear stability before the bifurcation. This type of simple model is commonly used in dynamical systems literature, and a supercritical pitchfork bifurcation has specifically been used in an AMOC box model (Dijkstra and Molemaker, 1997). These models, however, are generally intended to show how (new) statistics work in a ‘not very challenging’ environment, rather than be perfect analogues for climate systems.

We can, however, design more challenging synthetic systems to test our new method on. If we model, for example, a subcritical pitchfork, we can still use the same early-warning statistics and discover decreases in λ before a system state change (Figure 1). We can also use an AMOC box model with a drifting state (Figure 2) or a non-stationary fold which has a drifting state (x) term (Figure 3). In each case, our λ is a viable early-warning signal. We hence maintain that our work is not limited by the simplicity or ease of the synthetic model chosen for illustrative purposes, and the method we propose can be used in a wide array of cases.

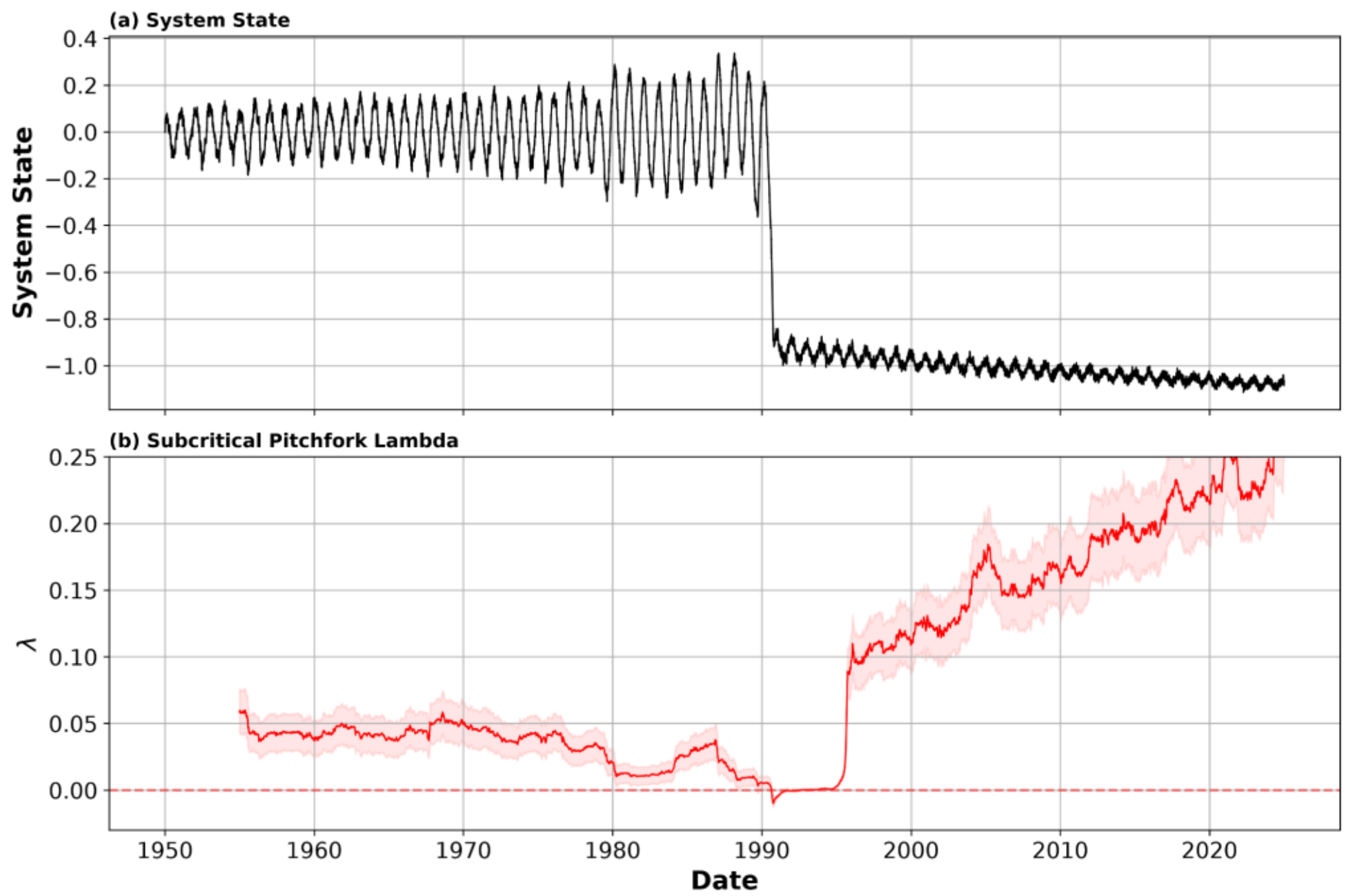

Figure 1: A subcritical pitchfork model defined as $dx = (\mu(t)x + x^3 - x^5 + A\cos(\omega t))\,dt + \sigma\,dW\_t$.

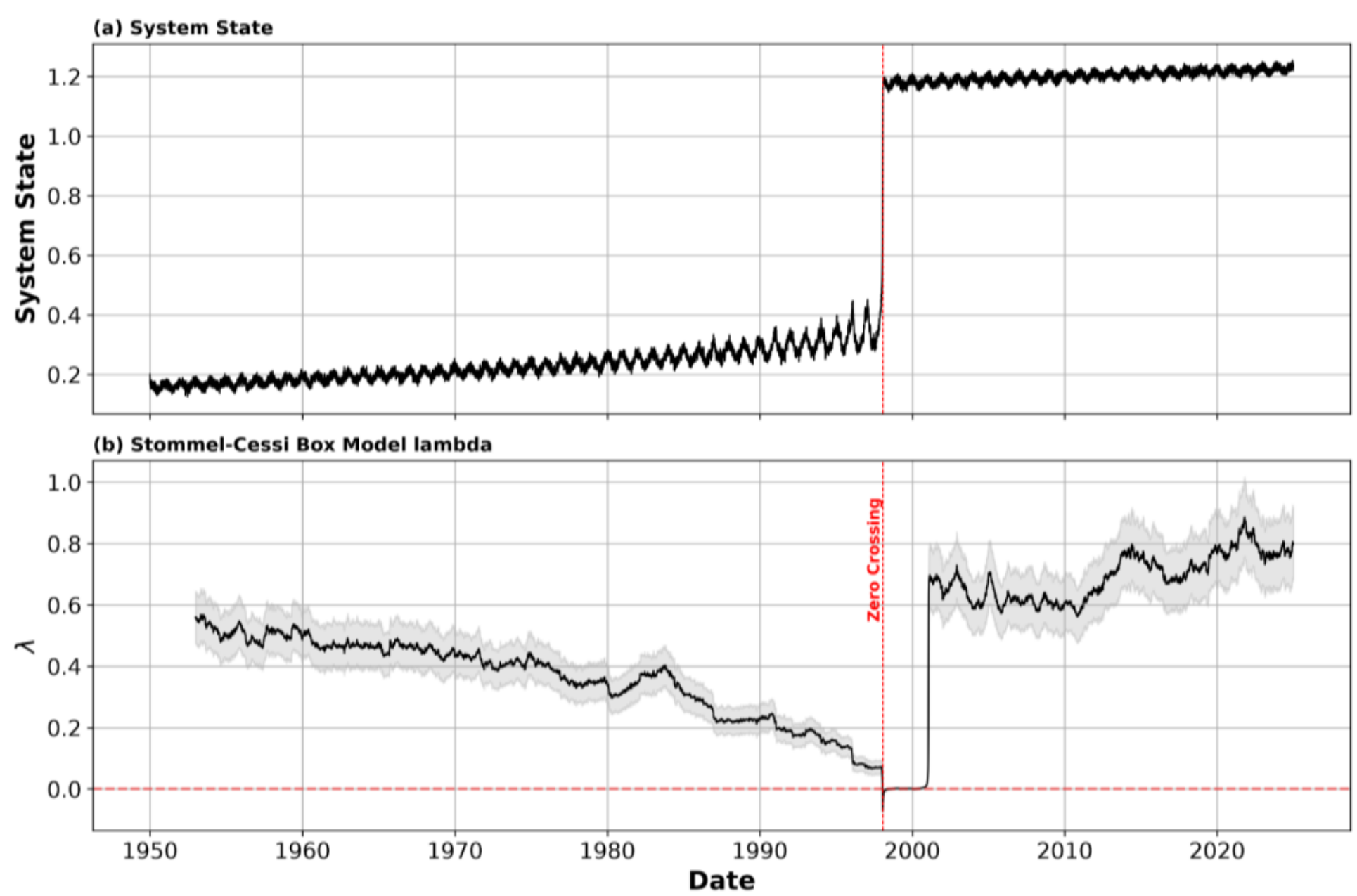

Figure 2: A Stommel-Cessi AMOC box model (Cessi, 1994; Lux-Gottschalk and Ritchie, 2025), defined as $dx = (\mu(t) - x(1 + \eta^2(1 - x)^2) + A\cos(\omega t))\,dt + \sigma\,dW\_t$.

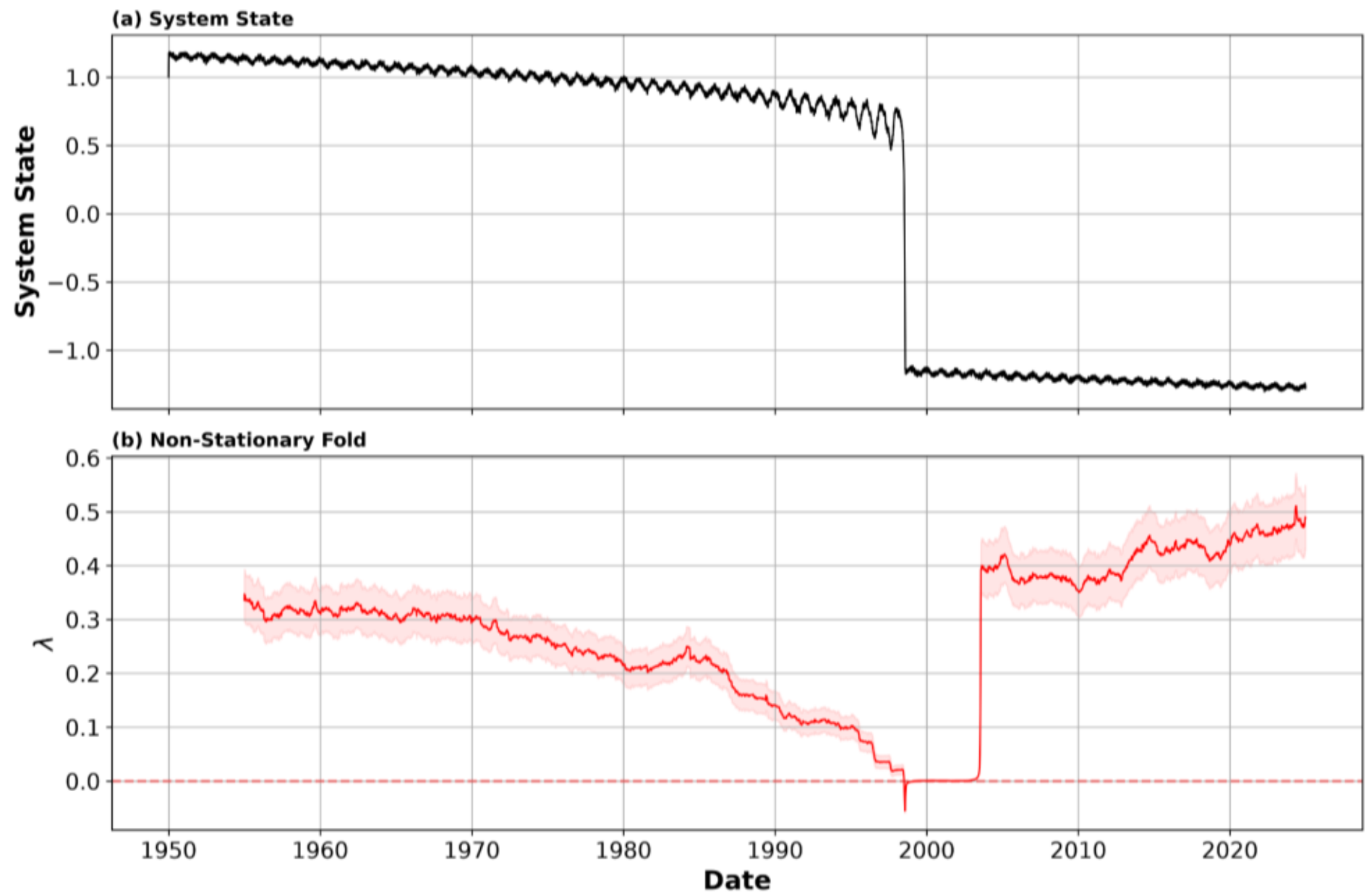

Figure 3: Non-stationary fold model, defined as $dx = (\mu(t) + x - x^3 + A\cos(\omega t))\,dt + \sigma\,dW_t$.

(d) I am concerned that even in the "basic model" the authors can't seem to get good agreement between parametric and non-parametric estimations of lambda: see Figure S8, which shows a similar trend, but the timescales are different by a factor of more than ten.

Thank you for this comment. We do not find this to be a surprising result, given that non-parametric estimators are difficult to correctly parameterize for many systems. They have a tendency to lead to signal leakage, often taking out too much or too little of the 'background' signal if the bandwidth is not perfectly aligned with the dynamics (and noise). We do not think that the non-parametric approach is right for the systems we are studying here – seasonal signals with a clearly modelable sinusoidal forcing. We include the non-parametric approach here for completeness, and to make it clear that our general regression framework would also be applicable to systems where the forcing is much harder to model. In such a case, non-parametric regression may be a better – or indeed the only – way to apply our Langevin regression approach.

We have added a further note explaining this difference to the Methods section where we describe the non-parametric approach.

(e) The captions should consistently indicate which system/data is being explored. In particular in the supplementary info.

We have updated the Figure captions in both the MS and Supplement to make this clearer.

Minor edits:

Supplementary equation (1) is described as “a saddle node bifurcation (cubic fold)” which suggests they are synonyms. They are not.

We used the parentheses here to note the system type which is used to express the bifurcation, not to state their equivalence. We have updated this text to be clearer.

In summary, I still recommend publication, but I am less sure of the significance of this paper.

Thank you for your comments in this and the previous round, as they have helped us better position and clarify our arguments.

References

Cessi, Paola. "A simple box model of stochastically forced thermohaline flow." *Journal of physical oceanography* 24.9 (1994): 1911-1920.

Dijkstra, Henk A., and M. Jeroen Molemaker. "Symmetry breaking and overturning oscillations in thermohaline-driven flows." *Journal of Fluid Mechanics* 331 (1997): 169-198.

Lux-Gottschalk, Kerstin, and Paul DL Ritchie. "Uncertainty quantification for overshoots of tipping thresholds." *Earth System Dynamics* 16.4 (2025): 1153-1168.

Reviewer #2:

The authors have greatly improved this paper, particularly with the extra analyses in the Methods section to discuss the limitations of the model. The non-parametric regression is particularly important for general application of the method.

Reviewer #2 (Remarks on code availability):

I have not reviewed the updated repository but was satisfied with the original code.

Thank you for taking the time to re-review the Manuscript, and we are glad to hear that our revisions have improved the work.

Reviewer #3:

The authors have made a commendable effort to thoroughly address my previous comments. The addition of several supplemental figures and the comprehensive sensitivity analyses regarding window length, noise structures, and model misspecification have effectively dealt with the concerns I raised. The manuscript is now significantly more robust, and the results are presented with a high degree of transparency.

I am pleased to recommend the revised version of this manuscript for publication in Nature Communications.

Thank you for your comments and detailed revisions! They were very helpful in improving our Manuscript.

I have only two minor points that the authors should consider for the final version:

Markov Assumption (Mandatory): In the theoretical framing of the methodology, it is necessary to explicitly state that by employing a Langevin-type equation to model the system dynamics, the state variable x(t) is assumed to be a Markov process. This is a fundamental underlying assumption of the framework that should be clear to the reader.

Thank you for this comment. This is indeed a good point to make clearer, and we have added this to the start of the Theoretical Framing section.

Estimation of Measurement Noise (Suggestion): As a constructive suggestion for their current or future work, the authors could note that measurement noise can be specifically estimated and separated from process noise using the approach presented in Phys. Rev. Lett. 97, 090603 (2006). Referencing this could provide additional guidance to researchers applying this method to highly noisy observational datasets.

Thank you for pointing out this interesting paper. We have added it as a reference to the updated Manuscript, and make a note about noise separation in our Methods section where we discuss the ratio of process to measurement noise.

Congratulations to all authors on a very high-quality and impactful contribution to the field.

Referee Report on "Estimating the Resilience of Non-Stationary Systems"

Authors: Taylor Smith, Andreas Morr, Christof Schötz, Niklas Boers

This manuscript addresses an important limitation in the estimation of resilience and critical slowing down (CSD) indicators from observational data. Traditional approaches based on lag-1 autocorrelation (AC1), variance, and related metrics generally rely on assumptions of stationarity, regular temporal sampling, and extensive preprocessing. These assumptions are often violated in Earth system applications, where trends, seasonality, irregular sampling, and missing observations are common.

The authors employ a regression-based framework derived from the Langevin equation that explicitly incorporates a time-dependent attractor. By representing the attractor through a design matrix (e.g., harmonic and polynomial basis functions), the method simultaneously estimates both the restoring rate (resilience parameter) and the attractor dynamics. The framework naturally accommodates irregular sampling and observational uncertainties.

The methodology is demonstrated on synthetic examples and subsequently applied to three important real-world cases: global vegetation resilience, NGRIP paleoclimate records, and glacier surge prediction. The manuscript is well written, mathematically correct, and addresses a timely and important problem in resilience estimation.

The manuscript makes several notable contributions. By moving from a sequential workflow-detrending followed by resilience estimation-to a simultaneous estimation framework, the approach reduces potential preprocessing biases. Its ability to handle irregularly sampled observations directly, without the need for interpolation, is particularly valuable for paleoclimate, remote sensing, and ecological datasets. In addition, the explicit propagation of observational and age-model uncertainties enhances the interpretability of the estimated resilience. Taken together, these advances make the framework a strong candidate for becoming a standard tool for assessing resilience in non-stationary Earth system records.

Major Comments

1. Sensitivity to Temporal Window Length

The proposed method relies on moving temporal windows to estimate local resilience. Although the authors discuss selecting window sizes according to the characteristic time scales of the system, a more systematic sensitivity analysis would strengthen the paper.

Specifically, it would be valuable to quantify how estimated restoring rates vary as a function of window length. Very short windows may lead to under-constrained regressions and larger uncertainties, whereas excessively long windows may violate the quasi-stationarity assumption underlying the local approximation.

Providing such an analysis for at least one synthetic example and one real-world application would help establish practical guidelines for future users.

2. Assumption Regarding Measurement Noise

In the derivation, the authors assume that process noise dominates measurement noise. While this assumption may be appropriate in many applications, it is not universally valid.

For several paleoclimate proxies and satellite-derived observations, measurement uncertainties can be substantial and may be comparable to process variability. I therefore encourage the authors to perform synthetic experiments in which the ratio between process noise and measurement noise is systematically varied.

This would clarify the regime in which the proposed estimator remains unbiased and provide guidance regarding its applicability to noisy datasets.

3. Attractor Model Misspecification

The framework requires the user to specify a functional representation of the attractor dynamics through the design matrix (e.g., Fourier components or polynomial trends).

A natural concern is whether misspecification of the attractor model introduces biases into the estimated restoring rate. For example, if the true attractor contains higher-frequency components or nonlinear structures not represented in the chosen basis, can these unmodeled components leak into the resilience estimate?

The authors should discuss this issue more explicitly and, if possible, provide a synthetic example illustrating the impact of attractor misspecification on the inferred restoring rate.

4. Cross-Validation via Kramers–Moyal Coefficients

The proposed regression framework can be interpreted as a parametric estimation of the first Kramers–Moyal coefficient $D^{(1)}$, corresponding to the deterministic drift term of the underlying stochastic process.

To further validate the methodology, I recommend that the authors compare their estimates with standard non-parametric Kramers–Moyal reconstruction techniques. In particular:

- Estimate the first Kramers–Moyal coefficient using the kernel-based approaches on synthetic datasets.
- Compare the local gradient of the reconstructed drift function with the restoring rates obtained from the regression framework.
- Demonstrate consistency between the parametric and non-parametric estimates.

Such a comparison would establish a direct connection between the proposed method and the classical stochastic-process framework underlying Langevin dynamics.

5. Non-Parametric Benchmarking Using Kernel Regression

The methodology relies on a predefined representation of the time-varying attractor. Although the authors demonstrate robustness for several examples, it remains unclear how sensitive the results are to the specific basis functions chosen.

As a robustness check, I suggest employing a non-parametric benchmark such as Nadaraya–Watson kernel regression or local linear regression to estimate the evolving attractor.

The resulting resilience estimates could then be compared with those obtained from the proposed harmonic or polynomial design matrices. Agreement between the two approaches would substantially strengthen confidence that the inferred resilience trends are not artifacts of the chosen functional form.

6. Comparison with Alternative Reconstruction Frameworks

The global vegetation application represents a high-dimensional spatio-temporal system in which spatial interactions and network effects may play a role.

For this reason, it would be informative to compare the reported resilience trends with those obtained using an alternative dynamical reconstruction framework such as HINTS (HiNTS: Higher-Order Interactions in N-Dimensional Time Series) or related moment-based reconstruction techniques.

While a full analysis may be beyond the scope of the current manuscript, even a limited comparison on a subset of the vegetation dataset would provide an independent validation of the inferred resilience loss and demonstrate consistency across reconstruction methodologies.

7. Robustness to Noise Structure

The derivation assumes Gaussian white noise forcing. However, many Earth system records exhibit temporal correlations, heavy-tailed fluctuations, or state-dependent noise.

I encourage the authors to discuss the extent to which their restoring-rate estimates are affected by:

- temporally correlated (red) noise,
- non-Gaussian forcing,
- multiplicative or state-dependent noise.

Examining the behavior of the second Kramers–Moyal coefficient $D^{(2)}$ in the real-world examples may help determine whether diffusion effects influence the reported resilience estimates.

Such analyses would significantly strengthen the generality and robustness of the proposed framework.

Minor Comment

1. A brief discussion of computational efficiency would be useful. Although the proposed method is clearly more sophisticated than standard AC1 estimation, readers working with large spatio-temporal datasets would benefit from an indication of computational scaling and runtime.

Recommendation

Recommendation: Accept after Minor Revisions.

The requested revisions primarily concern additional validation, robustness analyses, and clarification of methodological assumptions. These suggestions are intended to further strengthen an already strong contribution rather than to address fundamental shortcomings.
